# Preoperative Prediction of Ki-67 Labeling Index By Three-dimensional CT Image Parameters for Differential Diagnosis Of Ground-Glass Opacity (GGO)

**DOI:** 10.1371/journal.pone.0129206

**Published:** 2015-06-10

**Authors:** Mingzheng Peng, Fei Peng, Chengzhong Zhang, Qingguo Wang, Zhao Li, Haiyang Hu, Sida Liu, Binbin Xu, Wenzhuo Zhu, Yudong Han, Qiang Lin

**Affiliations:** 1 Department of Thoracic Surgery, Shanghai First People’s Hospital Affiliated to The Shanghai Jiao Tong University School Of Medicine, Shanghai, China; 2 Department of Nephrology, People's Hospital of Hunan Province Affiliated to Hunan Normal University School Of Medicine, Changsha, Hunan Province, China; 3 Department of Radiology, Shanghai First People’s Hospital Affiliated to The Shanghai Jiao Tong University School Of Medicine, Shanghai, China; Memorial Sloan-Kettering Cancer Center, UNITED STATES

## Abstract

The aim of this study was to predict Ki-67 labeling index (LI) preoperatively by three-dimensional (3D) CT image parameters for pathologic assessment of GGO nodules. Diameter, total volume (TV), the maximum CT number (MAX), average CT number (AVG) and standard deviation of CT number within the whole GGO nodule (STD) were measured by 3D CT workstation. By detection of immunohistochemistry and Image Software Pro Plus 6.0, different Ki-67 LI were measured and statistically analyzed among preinvasive adenocarcinoma (PIA), minimally invasive adenocarcinoma (MIA) and invasive adenocarcinoma (IAC). Receiver operating characteristic (ROC) curve, Spearman correlation analysis and multiple linear regression analysis with cross-validation were performed to further research a quantitative correlation between Ki-67 labeling index and radiological parameters. Diameter, TV, MAX, AVG and STD increased along with PIA, MIA and IAC significantly and consecutively. In the multiple linear regression model by a stepwise way, we obtained an equation: prediction of Ki-67 LI=0.022*STD+0.001* TV+2.137 (R=0.595, R’s square=0.354, p<0.001), which can predict Ki-67 LI as a proliferative marker preoperatively. Diameter, TV, MAX, AVG and STD could discriminate pathologic categories of GGO nodules significantly. Ki-67 LI of early lung adenocarcinoma presenting GGO can be predicted by radiologic parameters based on 3D CT for differential diagnosis.

## Introduction

Advances in high resolution CT (HRCT) scanning had increased the detection of ground-glass opacity (GGO) with data from many studies suggesting that localized GGO represents as a precursor of lung adenocarcinoma.[1–5] Since a new international multidisciplinary classification of lung adenocarcinoma had been proposed by International Association for the Study of Lung Cancer (IASLC), the American Thoracic Society (ATS), and the European Respiratory Society (ERS) in 2011, pathologic differentiation of GGO has been imperative and attractive for thoracic radiologists and surgeons.[6]

GGO is a finding on HRCT lung images, and has also been described as a hazy increase in lung attenuation without obscuring the underlying bronchial or vascular structures.[3, 7, 8] Furthermore, lesions without solid component in it were classified to pure GGO in comparison to mix GGO with solid component and ground glass attenuation in it as well. As a nonspecific finding that indicates a variety of disorders, it is generally difficult to differentiate with only two-dimensional CT image features, even at follow-up. [3, 9, 10] Advances in understanding of pathologic and radiologic features of GGO have led to changes in diagnostic and therapeutic strategies.[11] Especially, an awareness of the significance of CT attenuation number in assessing GGO has been reported recently.[12–14] Three-dimensional (3D) evaluation has been shown to be more sensitive and precise for quantifying small pulmonary nodules, particularly for asymmetric nodules, than one- or two-dimensional methods.[15, 16]

Besides, proliferation is a key feature for progressing of lung cancer, which is now widely estimated by the immunohistochemical assessment of the nuclear antigen Ki-67. Some authors[17–21] have demonstrated that proliferative activities determined by Ki-67 were correlated with the prognosis of lung cancer patients.

Thus, at this present study, we combined more objective and accuracy parameters obtained from 3D CT image and Ki-67 labeling index (LI) of GGO to analyze their correlation quantitatively and unprecedentedly to predict Ki-67 LI by 3D CT image parameters for preoperative assessment.

## Materials and Methods

This study was reviewed and approved by Institutional Review Board of Shanghai First People’s Hospital with Certificate Number of 2014KY115 and written informed consent for patients to participate in this research was obtained before the retrospective study.

### Patients

All of our selected cases were adenocarcinoma that recently has been the most frequent pathologic type of lung cancer.[22] All those patients detected out a GGO lesion by HRCT should have been treated by antibiotic for two weeks or so at first. Another CT scan would be performed after at least three months as a follow-up. Only the stable or size-increasing lesions after anti-inflammatory could be selected into this research with excluding those transient ones reckoned as inflammatory in most cases.[23] Only pure and mix focal GGO (part-solid) nodules with diameters less than 3 cm were included. Henschke et al reported that GGO nodules have a higher malignancy rate than full-solid nodules.[24] Feng Li et al also found that there were only 15 malignancy lesions in comparison to 122 benign ones in all 137 cases presenting small full-solid nodules on CT screen. Among full-solid nodules, a polygonal shape or a smooth or somewhat smooth margin was present less frequently in malignant than in benign lesions (polygonal shape: 7% vs. 38%, *P* = 0.02; smooth or somewhat smooth margin: 0% vs. 63%, *P* = 0.001), and 98% (46 of 47) of polygonal nodules and 100% (77 of 77) of nodules with a smooth or somewhat smooth margin were benign.[25] Hence, we did not include small full-solid nodules as GGO component attractive more our attention as well as other researchers’.

The exclusion criteria were as follows: (1) cases without pathologic diagnosis or preoperative CT scanning in our hospital, (2) mean diameters of three axes larger than 3 cm, (3) small cell lung cancer, squamous carcinoma and metastatic carcinoma, (4) adenocarcinoma exceed T1N0M0, (5) cases with chemo-radiotherapy or biopsy preoperatively, (6) cases with a history of previous primary lung cancer or extra-pulmonary malignancy. Eventually, 160 patients (54 men and 106 women, mean age 56.59±9.9 years) undergoing HRCT examinations preoperatively were selected. The interval between the latest preoperative HRCT and surgery was 11±4 days with range of 1 to 23 days. Pathologic diagnoses included atypical adenomatous hyperplasia (AAH, n = 26), adenocarcinoma in situ (AIS, n = 11), minimally invasive adenocarcinoma (MIA, n = 106) and invasive adenocarcinoma (IAC, n = 17). Table 1 shows the clinical, radiologic and pathologic characteristics of the selected cases.

**Table 1:** Clinical, Radiologic and Pathologic Characteristics of All GGO Nodules with different pathologic categories (n = 160).

**Variables**	**PIA (n = 37)**	**MIS (n = 106)**	**IAC (n = 17)**	**P**	**P1**	**P2**
****Gender (NO.)****				0.027*	0.023*	0.593
****Male****	6	40	8			
****Female****	31	66	9			
****Mean age (years)****	53.05±8.12	56.69±10.28	62.94±7.57	0.002*	0.032*	0.018*
****Smoking history (NO.)****				0.576	0.447	0.639
****Positive****	1	8	1			
****Negative****	36	98	16			
****GGO subtype****				0.003*	0.015*	0.072
****Pure GGO****	18	28	1			
****Mix GGO****	19	78	16			
****GGO number****				0.241	0.698	0.17
****Solitary****	22	67	14			
****Multiple****	15	39	3			
****Position****				0.692	0.375	0.851
****RUL****	16	42	8			
****RML****	4	15	1			
****RLL****	6	8	1			
****LUL****	7	33	5			
****LLL****	4	8	2			
****3D CT Image Parameters****						
****Diameter (mm)****	8.65±3.09	14.68±5.81	20.17±6.15	<0.001*	<0.001*	<0.001*
****Total volume (TV) (mm**^**3**^**)****	167.527±133.2	819.73±874.71	1563.45±1200.59	<0.001*	0.01*	<0.001*
****Maximum CT number (MAX)(HU)****	-218.19±182.54	21.09±182.2	265.31±359.42	<0.001*	<0.001*	0.001*
****Average CT number (AVG)(HU)****	-656.62±48.26	-575±56.04	-506.62±103.21	<0.001*	<0.001*	0.001*
****Standard deviation of CT number within nodule (STD)****	88.30±28.65	155.13±41.83	187.32±61.91	<0.001*	<0.001*	0.031*
****Ki-67****						
****Ki-67 labeling index (LI) (%)****	2.71±1.27	6.24±1.66	9.72±2.24	<0.001*	<0.001*	<0.001*
****Prediction of Ki-67 labeling index (%)****	4.27±0.76	6.50±1.69	8.81±3.73	<0.001*	<0.001*	0.014*

Note:Classified according to the new IASLC/ATS/ERS International Multidisciplinary Lung Adenocarcinoma Classification system.

Unless otherwise indicated, numerical data were recorded as the mean ± standard deviation (SD).

RUL stands for Right Upper Lobe, RML stands for Right Middle Lobe, RLL stands for Right Low Lobe, LUL stands for Left Upper Lobe, and LLL stands for Left Low Lobe.

*P<0.05

Gender, smoking history, GGO subtype, GGO number and position were analyzed by Chi-square test.

Mean age, diameter, TV, MAX, AVG, STD and prediction of Ki-67 LI were analyzed by Kruskal-Wallis and Tamhane’s T2 test.

Ki-67 LI was analyzed by one-way ANOVA and Least Significant Difference (LSD) test.

P indicates the p values for one-way ANOVA or Kruskal-Wallis analyses of all GGO nodules.

P1 indicates the p values for LSD test or Tamhane’s T2 test of PIA versus MIA.

P2 indicates the p values for LSD test or Tamhane’s T2 test of MIA versus IAC.

### Image Acquisition

CT images were obtained from thoracic inlet to lung base with patients at full inspiration. No intravenous contrast material was injected. CT images were acquired by using CT scanners (Somatom Plus 4, Siemens, Erlangen, Germany; LightSpeed Ultra, GE Medical Systems, Milwaukee, Wis; or Mx8000, Philips Medical System, Andover, Mass). Tube voltage ranged from 120 to 140 kV, and tube current ranged from 200 to 400 mA. Axial images of 0.625 mm thickness with 0.5 mm spacing were reconstructed with 512×512 matrix by using bone algorithm axial reconstruction and filtered back projection (FBP) algorithm and the smallest field of view that included both lungs. Two chest radiologists, with 8 and 12 years’ experience of reading CT images of the chest respectively, identified diagnoses of GGO on CT images by consensus.

### Computerized Volumetry of GGO Nodules After 3D Measurement

**Fig 1:**
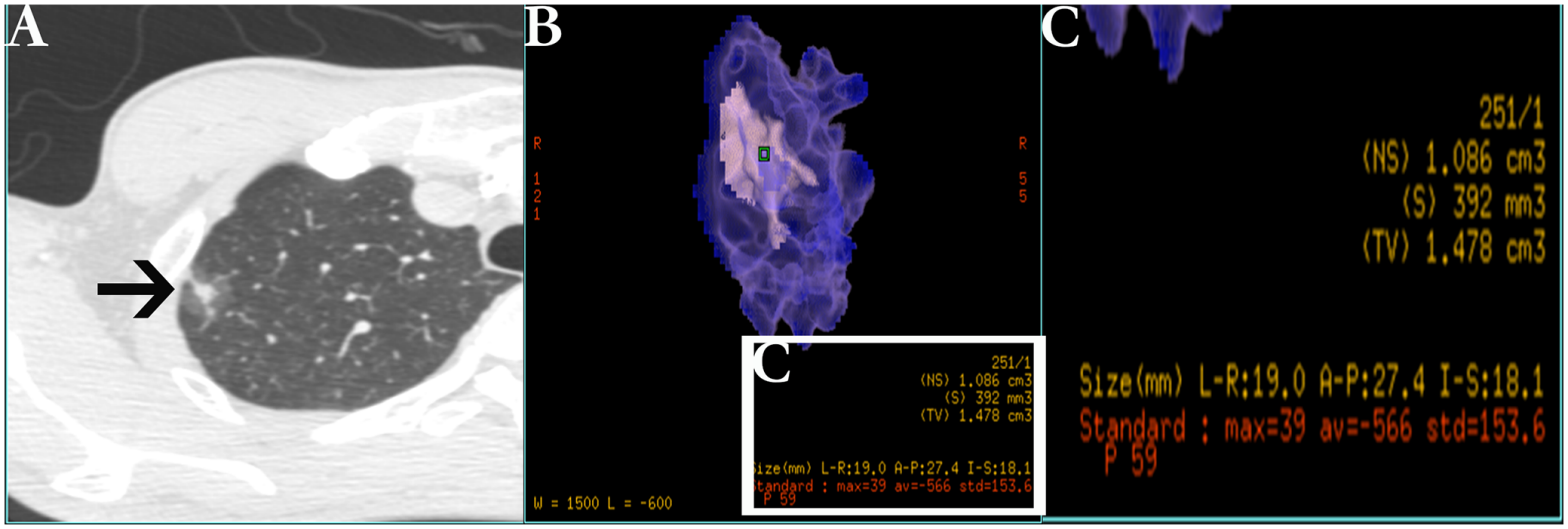
Example of nodule three-dimensional processed and measured on thin-section helical computed tomography (CT) images. (A) Typical ground-glass opacity (GGO, black arrow) nodule on high-resolution CT. (B) The software automatically processed and measured the nodule that be placed a marker (green square) on it. (C) The magnified measurement list of relative image parameters from picture B including diameter, total volume (TV), the maximum CT value (MAX), average CT value (AVG) and standard deviation of CT value (STD).

In the present study, we analyzed 5 radiologic parameters of GGO: diameter, total volume (TV), the maximum CT number (MAX), average CT number (AVG) and standard deviation of CT number within the whole GGO nodule (STD) that were measured on a commercially available workstation (Advantage Workstation 4.3; GE Healthcare) with CT lung analysis software (Lung VCAR; GE Healthcare). This software can segment pulmonary nodules with ground-glass attenuation. Diameter, TV, MAX, AVE and STD are computed automatically after the operator placing a marker on the nodule. The CT lung analysis software system automatically identified the GGO nodules in all X-axis, Y-axis and Z-axis directions from the surrounding normal lung tissue. The elimination of normal structures within or around the nodule, such as vessels and bronchiole, was performed using several image-processing techniques[26]. Therefore, the nodule was identified as the lesion area without vessels and bronchiole. Some authors have described the concrete procedures and methods with the exact kind of software.[27–31] The judgment of successful segmentation was based on the observers’ visual assessment on axial CT images as well as sagittal and coronal multiplanar reconstructed images. (Fig 1)

### Pathologic Diagnoses

**Fig 2:**
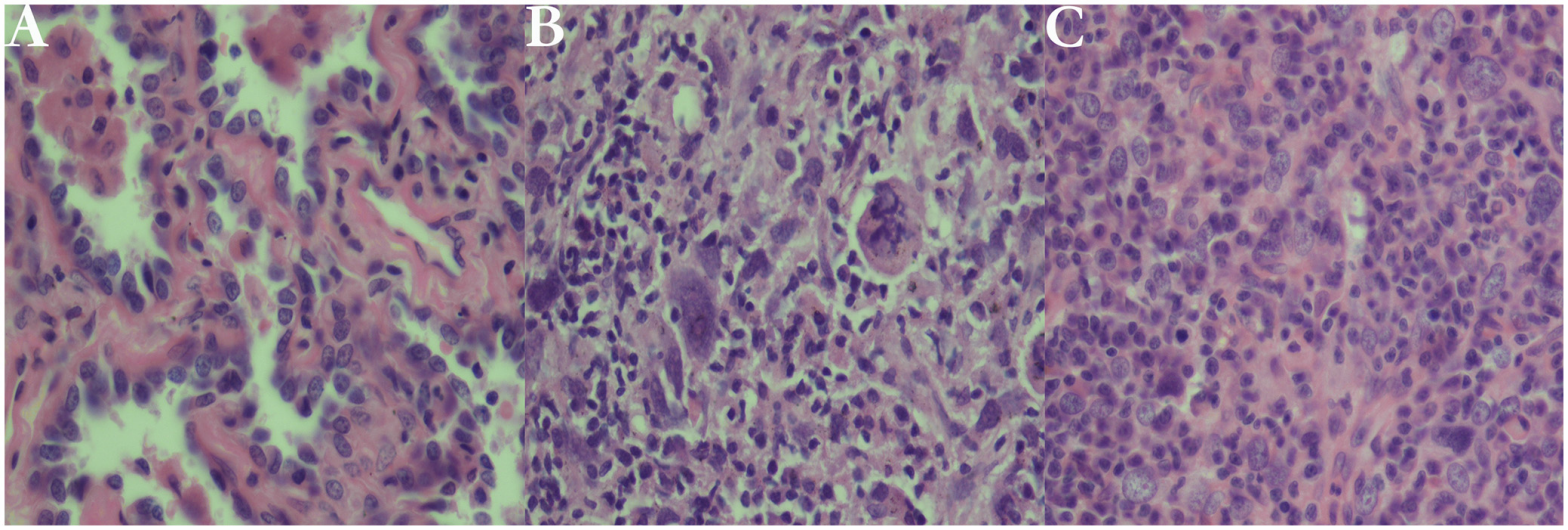
Photomicrographs of ground-glass opacity (GGO) (hematoxylin-eosin stain, original magnification, ×400). (A) A case of atypical adenomatous hyperplasia (AAH) with a well-defined boundary and pulmonary mesenchyme. (B) A case of adenocarcinoma in situ (AIS) with growth restricted to preexisting alveolar walls. No foci of invasion or scarring are seen. (C) A case of minimally invasive adenocarcinoma (MIA) consisting primarily of lepidic growth with a small area of acinar invasion.

The surgically resected specimens were routinely fixed in 10% formalin, and processed into paraffin blocks for pathologic examination over the entire volumes of GGO nodules. Tissue sections were cut at 4μm thickness, including the largest cut surface of the tumor, and stained with both hematoxylin and eosin (H&E). Pathologic diagnoses were made by two experienced lung pathologists. According to the new IASLC/ATS/ERS classification,[6] all of GGO nodules were diagnosed as AAH, AIS, MIA and IAC. (Fig 2)

### Detection of Ki-67 LI

**Fig 3:**
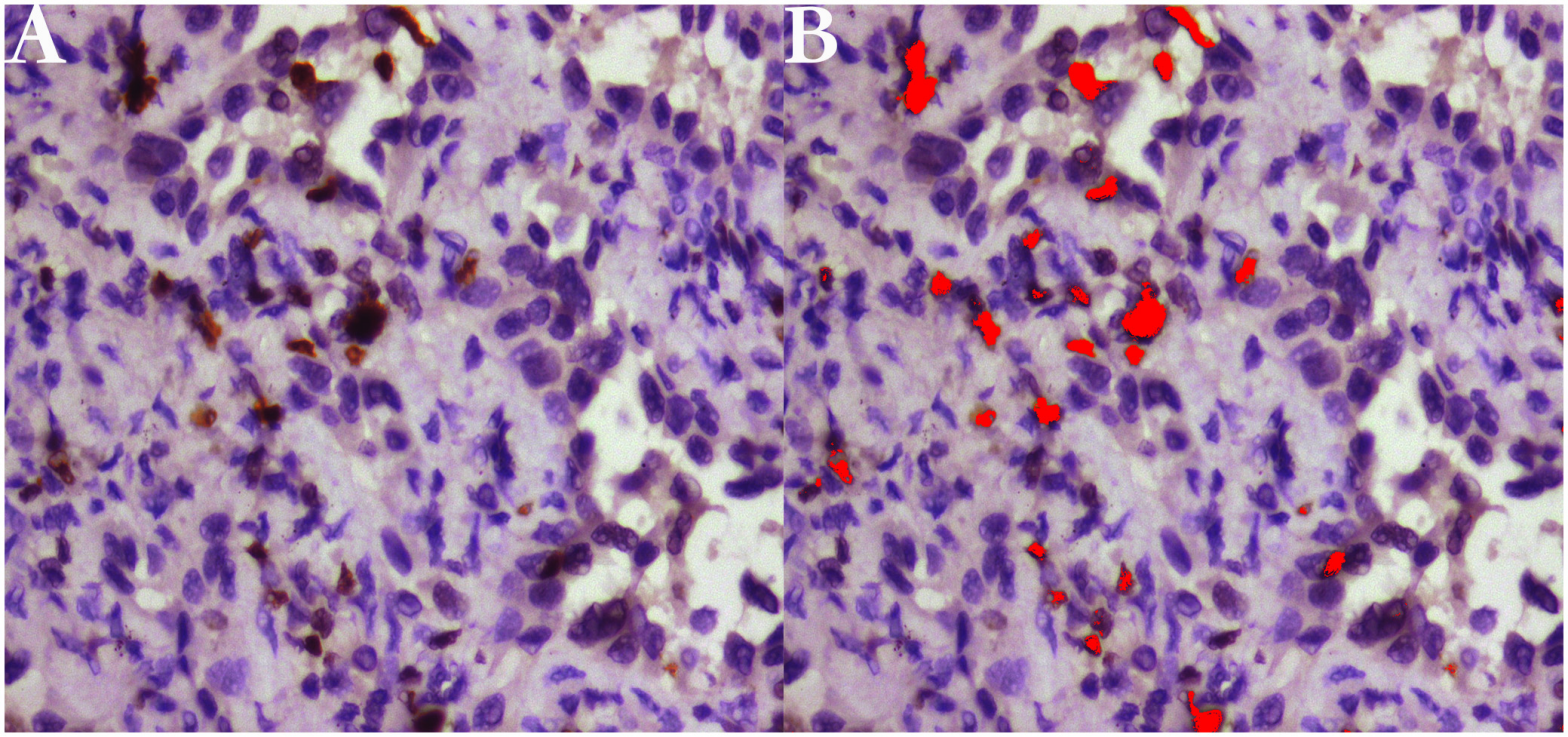
Representative immunohistochemical detection and measurement of Ki-67 labeling index (LI) (magnifyication ×400). (A) A case with positive expression of Ki-67 presenting tan particles located in nucleus of proliferating cells. (B) Quantitative measurement of Ki-67 LI by Image Software Pro Plus 6.0 (red area means detection of the software).

Immunostaining was performed with the standard streptavidin-perosidase (SP) technique with the antibodies for Ki-67 (monoclonal mouse antibody MIB-1, 1: 100 dilution). Representative formalin fixed, paraffin-embedded tissues were selected for immunohistochemistry. 4 μm thick tumor tissue sections were cut and dried for 20mins at 68°C. Routine deparaffinage by dimethylbenzene and dehydration by gradient ethyl alcohol was performed. 3% hydrogen peroxide was used to block endogenous peroxidase for 10mins at 37°C and phosphate buffer solution (PBS) was used to douche lasting 5mins for 3 times. Then citrate buffer with pH of 6.0 was used to repair antigen for 3mins. After that, sections were incubated with primary monoclonal mouse antibodies overnight at 4°C. The next step is put them into horseradish peroxidase complex (HRP) and diaminobenzidine (DAB) for 30mins and 10mins respectively at 37°C. Finally, routine hematoxylin redyeing, dehydration, dimethylbenzene transparentizing and gum mounting had been performed step by step. Quantitative analyses of positive expression of Ki-67 indicated by tan staining particles located in nucleus were performed using Image Software Pro Plus 6.0. Not only verified its accuracy by some authors,[32] this software has been applied to numerous aspects in biomedicine recently.[33–36] (Fig 3)

### Statistic Methods

Numerical data were recorded as mean ± standard deviation (SD). Levene test was performed for evaluating homogeneity of variance of variables. Consequently, variances of all variables other than that of Ki-67 LI were heterogeneous. (Table 2) Kruskal-Wallis test was taken for analyzing significant difference of variables with heterogeneous variance and analysis of variance (ANOVA) for Ki-67 LI with homogeneous variance among groups. Furthermore, we made Least Significant Difference (LSD) test for pairwise comparison of Ki-67 LI and Tamhane’s T2 for pairwise comparison of diameter, TV, MAX, AVG, STD and prediction of Ki-67 LI, whose variances were heterogeneous. In receiver operating curve (ROC) analysis, optimal cut-off points were defined as those on the curves closest to the upper left-hand corner[37]. Spearman correlation and multiple linear regression analysis with 10-fold cross-validation were performed to research the quantitative relation between Ki-67 LI and radiological parameters. All analyses were conducted with a statistical software package (SPSS, version 20.0). P-values of less than 0.05 were considered to indicate statistical significance.

**Table 2:** Levene test of variables for homogeneity of variance.

Variables	Levene Statistic	Sig.
Diameter	5.789	0.004
TV	20.413	<0.001
MAX	5.981	0.003
AVG	9.513	<0.001
STD	3.821	0.025
Ki-67 LI	1.888	0.156
Prediction	3.221	0.044

Note: variables include diameter of GGO nodules, total volume (TV), the maximum CT number (MAX), average CT number (AVG), standard deviation of CT number within the whole GGO nodule (STD), Ki-67 labeling index (LI) and prediction value of Ki-67 LI from multi-linear regression model.

*p* (Sig.)<0.05 means variance are heterogeneous,

*p* (Sig.)>0.05 means variance are homogeneous.

Variance of Diameter, TV, MAX, AVG, STD and Prediction were heterogeneous, which means they all would be tested by Tamhane’s T2 test. As for homogeneous variance, Ki-67 LI would be tested by Least Significant Difference (LSD) test for significant difference among groups.

## Results

### Radiological Features and Histological Diagnoses

**Fig 4:**
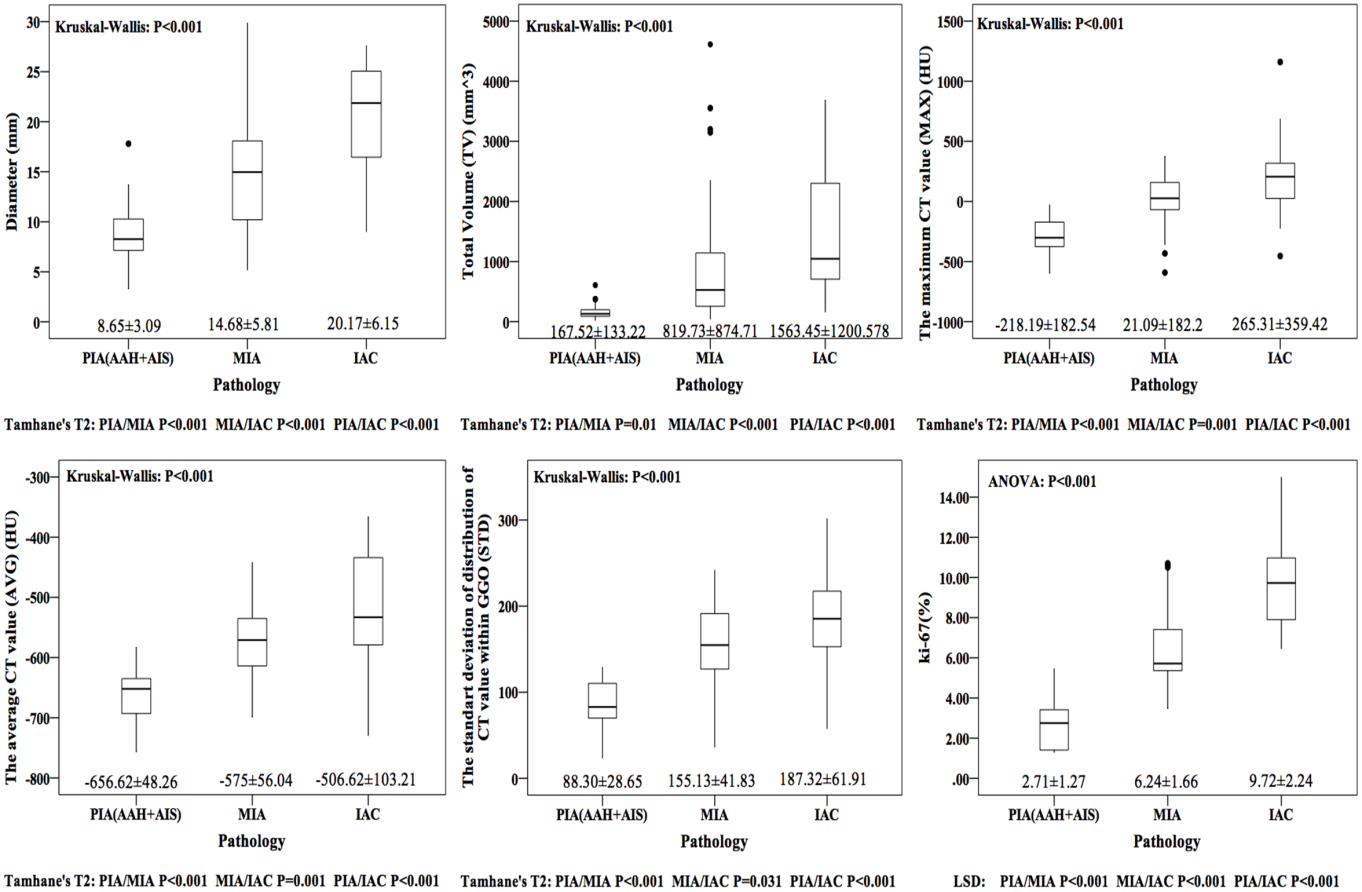
Graphs showing the mean diameter, total volume (TV), the maximum CT value (MAX), average CT value (AVG), standard deviation of CT value within the whole nodule (STD) and Ki-67 labeling index (LI) for preinvasive adenocarcinoma (PIA), minimally invasive adenocarcinoma (MIA) and invasive adenocarcinoma (IAC). Ki-67 LI was analyzed by ANOVA and LSD test due to homogeneity of variance and other variables were analyzed by Kurskal-Wallis test and Tamhane’s T2 for heterogeneity of variance. While all variables were significantly different among PIA, MIA and IAC.

Parameters of GGO were automatically calculated and displayed on the multi-display CT (MDCT) after 3D measurement by the CT lung analysis software. (Fig 4) The positive expression of Ki-67 presented tan particles located in nucleus of over proliferative cell. All of relevant data were recorded in Table 1.

### ROC Analysis for Pathologically Discrimination

**Fig 5:**
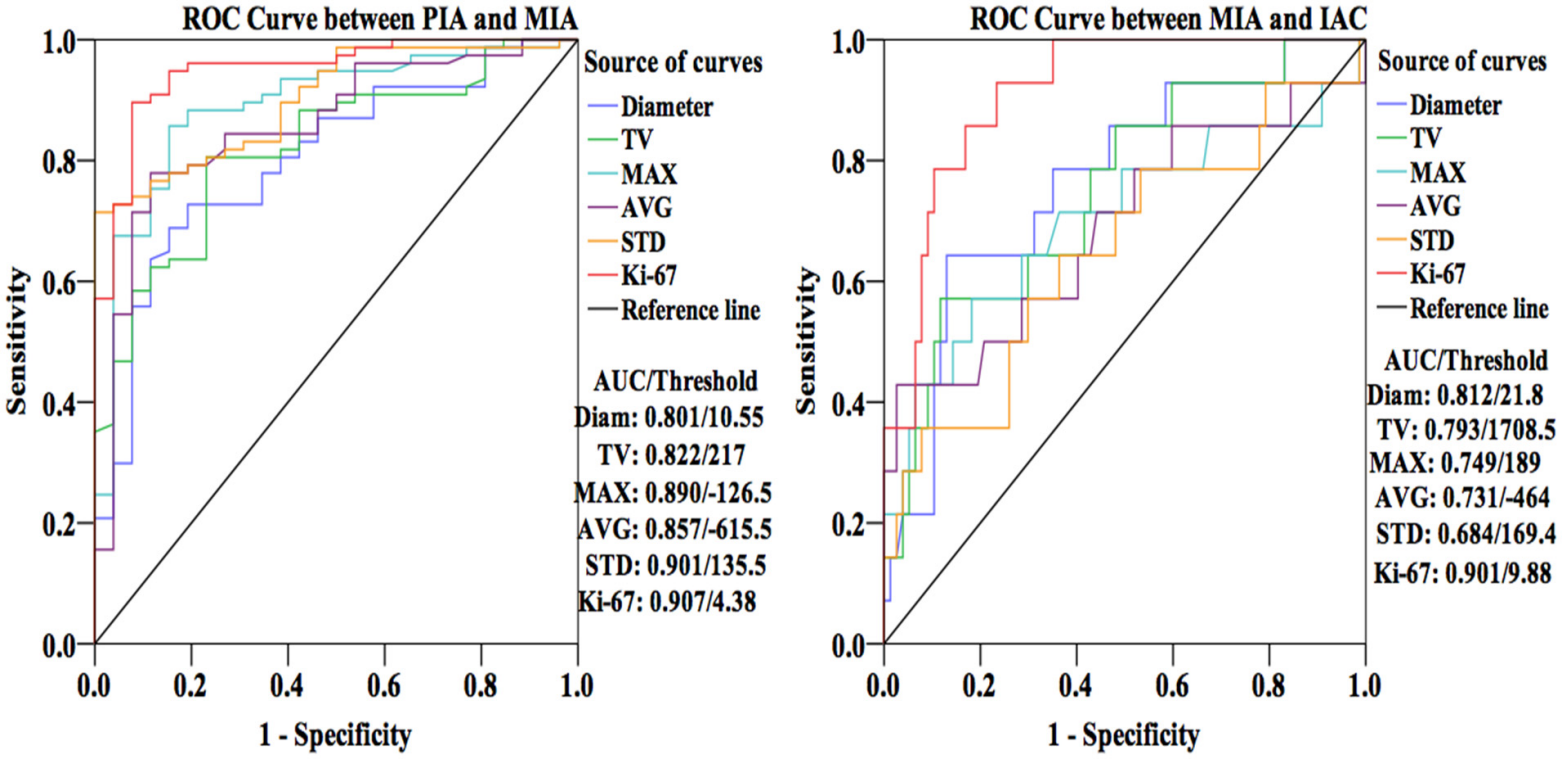
Receiver operating characteristic (ROC) curves of diameter, total volume (TV), the maximum CT value (MAX), average CT value (AVG), standard deviation of CT value within the whole nodule (STD) and Ki-67 labeling index (LI) for differentiation among PIA, MIA and IAC. According to area under the curve (AUC) of all variables, Ki-67 LI had a better differential capability than other parameters. It is easier to differential diagnose between PIA and MIA than between MIA and IAC.

By ROC analysis, area under the curve (AUC) of diameter, TV, MAX, AVG, STD and Ki-67 LI is 0.801, 0.822, 0.890, 0.857, 0.901 and 0.907 respectively to differentiate PIA from MIA and 0.812, 0.793, 0.749, 0.731, 0.684 and 0.901 respectively to rule out MIA from IAC. When it comes to the maximum true-positive ratio (sensitivity) and the minimum false-positive ratio (1—specificity) simultaneously for parameters, we obtained the thresholds of all variables. The optimal cut-off point of diameter, TV, MAX, AVG, STD and Ki-67 LI is 10.55 mm, 217 mm^3^, -126.5 HU, -615.5 HU, 135.5 and 4.38% respectively for the comparison between PIA and MIA. Corresponding to discrimination between MIA and IAC, the thresholds were 21.8 mm, 1708.5 mm^3^, 189 HU, -464 HU, 169.4 and 9.88% for diameter, TV, MAX, AVG, STD and Ki-67 LI respectively. (Fig 5)

### Correlation between 3D Image Parameters and Ki-67 LI

**Fig 6:**
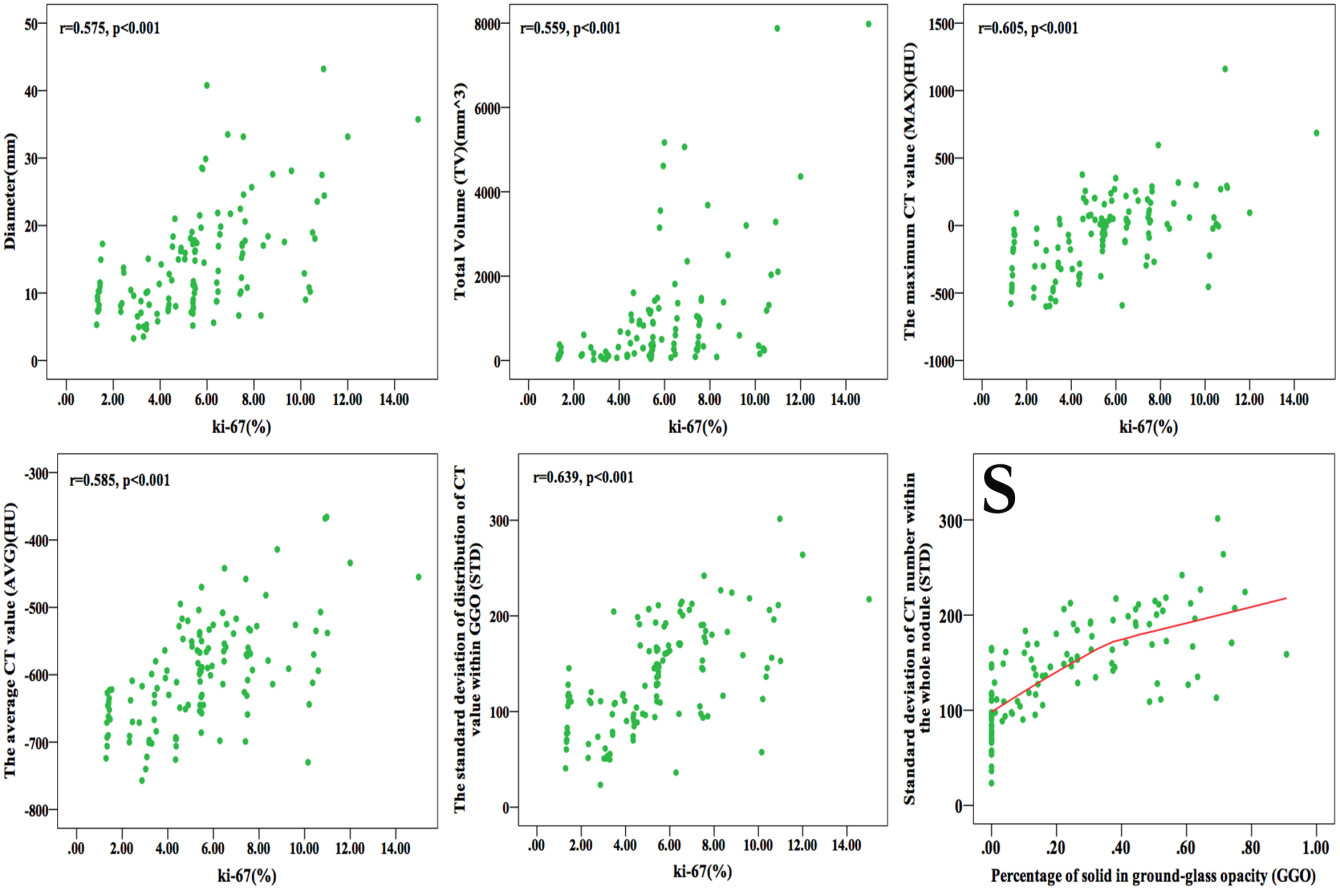
Graphs showing the correlation between Ki-67 labeling index (LI) and diameter, total volume (TV), the maximum CT value (MAX), average CT value (AVG), standard deviation of CT value within the whole nodule (STD) respectively, and correlation between STD and percentage of solid component in ground-glass opacity (GGO) nodule. (r means correlative coefficient) Diameter, TV, MAX, AVG and STD were positive correlation with Ki-67. Graph (S) shows that in the heterogeneous model of GGO, STD increase along with solid component growing in it instead of decrease after solid-part more than 50% of nodule.

Based on Spearman correlative analysis to Ki-67 LI, the correlative coefficient was 0.575, 0.559, 0.605, 0.585 and 0.639 for diameter (p<0.001), TV (p<0.001), MAX (p<0.001), AVG (p<0.001) and STD (p<0.001) respectively. (Fig 6)

Furthermore, multiple linear regression analysis was performed to quantitatively explore the relation between 3D parameters and Ki-67 LI. We also made a 10-fold cross-validation. After dividing 160 cases into 10 equal groups randomly, we defined 1 group including 10 cases as testing set and the other 9 groups as training set every time by turns. Next, we established 10 multi-linear regression models by training sets by a stepwise way and then put respective testing sets into its model to get 10 predictive value of Ki-67 every time. In every model, we calculated mean absolute error (MAE), mean relative error (MRE), and root mean square error (RMSE) for evaluating accuracy of equations obtained above. (Table 3)

**Table 3:** 10-fold cross-validation of multi-linear regression model for Ki-67 by CT image parameters of 160 cases.

Model No.	R	R^**2**^	MAE	MRE	RMSE	***p***	Equation
1	0.592	0.315	1.192	0.357	1.874	<0.001	Ki-67 LI = 0.019*STD+0.001*TV+2.432
2	0.571	0.326	1.534	0.332	1.811	<0.001	Ki-67 LI = 0.019*STD+0.001*TV+2.603
3	0.623	0.388	3.021	0.617	3.330	<0.001	Ki-67 LI = 0.02*STD+0.001*TV+2.269
4	0.614	0.376	2.157	0.711	3.326	<0.001	Ki-67 LI = 0.022*STD+0.001*TV+2.147
****5****	**0.595**	**0.354**	**1.052**	**0.178**	**1.399**	**<0.001**	Ki-67 LI = 0.022*STD+0.001*TV+2.137
6	0.585	0.343	2.079	0.469	2.635	<0.001	Ki-67 LI = 0.02*STD+2.348
7	0.593	0.351	1.379	0.350	2.123	<0.001	Ki-67 LI = 0.019*STD+0.001*TV+2.385
8	0.557	0.31	1.167	0.412	1.426	<0.001	Ki-67 LI = 0.017*STD+0.001*TV+2.796
9	0.519	0.27	2.365	1.003	2.705	<0.001	Ki-67 LI = 0.017*STD+0.001*TV+2.96
10	0.529	0.28	1.731	0.248	3.062	<0.001	Ki-67 LI = 0.021*STD+2.37

Note: R means multiple relative coefficient, R^2^ means determinate coefficient, MAE means mean absolute error, MRE means mean relative error and RMSE means root mean square error.

R and R^2^ reflect fitting degree of regression models.

MAE, MRE and RMSE reflect predictive accuracy of regression models. The lower MAE, MRE and RMSE are, the higher the predictive accuracy of regression model is.

$$MAE=\frac{1}{n}\;\sum_{i=1}^{n}\left|{\hat{y}}_{i}-y_{i}\right|$$ (1)

$$MRE=\frac{1}{n}\;\sum_{i=1}^{n}\left|\frac{{\hat{y}}_{i}-y_{i}}{y_{i}}\right|$$ (2)

$$\;RMSE=\sqrt{\frac{1}{n}\;\sum_{i=1}^{n}{\left({\hat{y}}_{i}-y_{i}\right)}^{2}}$$ (3)

In above formulas, n means number of cases in testing set, ${\hat{y}}_{i}$ means predictive value of Ki-67 LI, and $y_{i}$ means actual measured Ki-67 LI.

Among 10 regression equations, No. 5 owns higher predictive accuracy than others in spite that it was not the best fitting one. As a result of cross-validation, we established a more accurate regression model for predicting Ki-67 LI by CT image parameters based on three-dimensional process, stating as follows:

Ki−67LI=0.022*STD+0.001*TV+2.137 (4)

**Fig 7:**
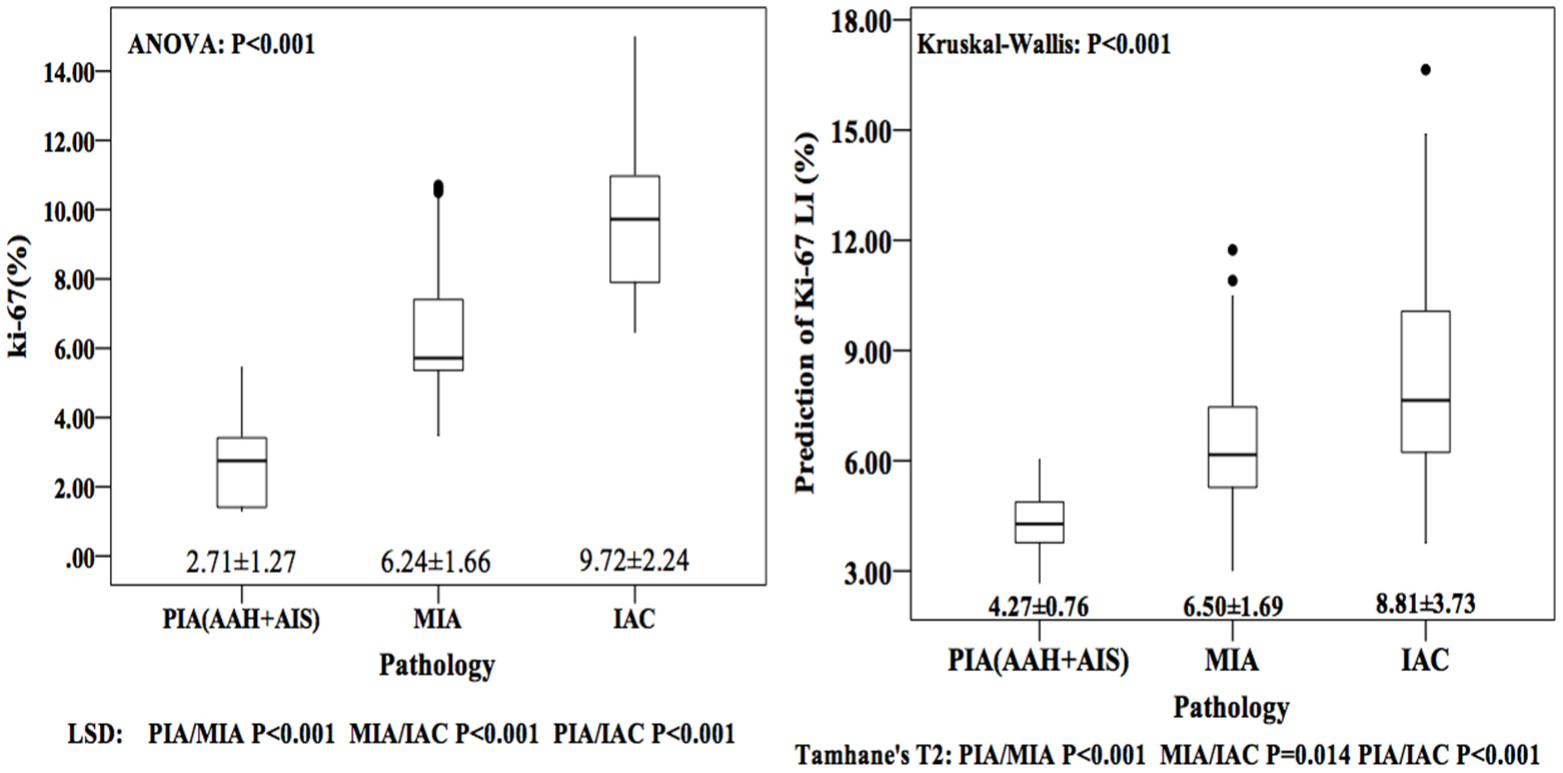
Boxplots comparing the mean predictive Ki-67 labeling index (LI) with mean actual measured Ki-67 LI of preinvasive adenocarcinoma (PIA), minimally invasive adenocarcinoma (MIA) and invasive adenocarcinoma (IAC). Predictive Ki-67 LI and actual measured Ki-67 LI were all significantly different among PIA, MIA and IAC.

**Fig 8:**
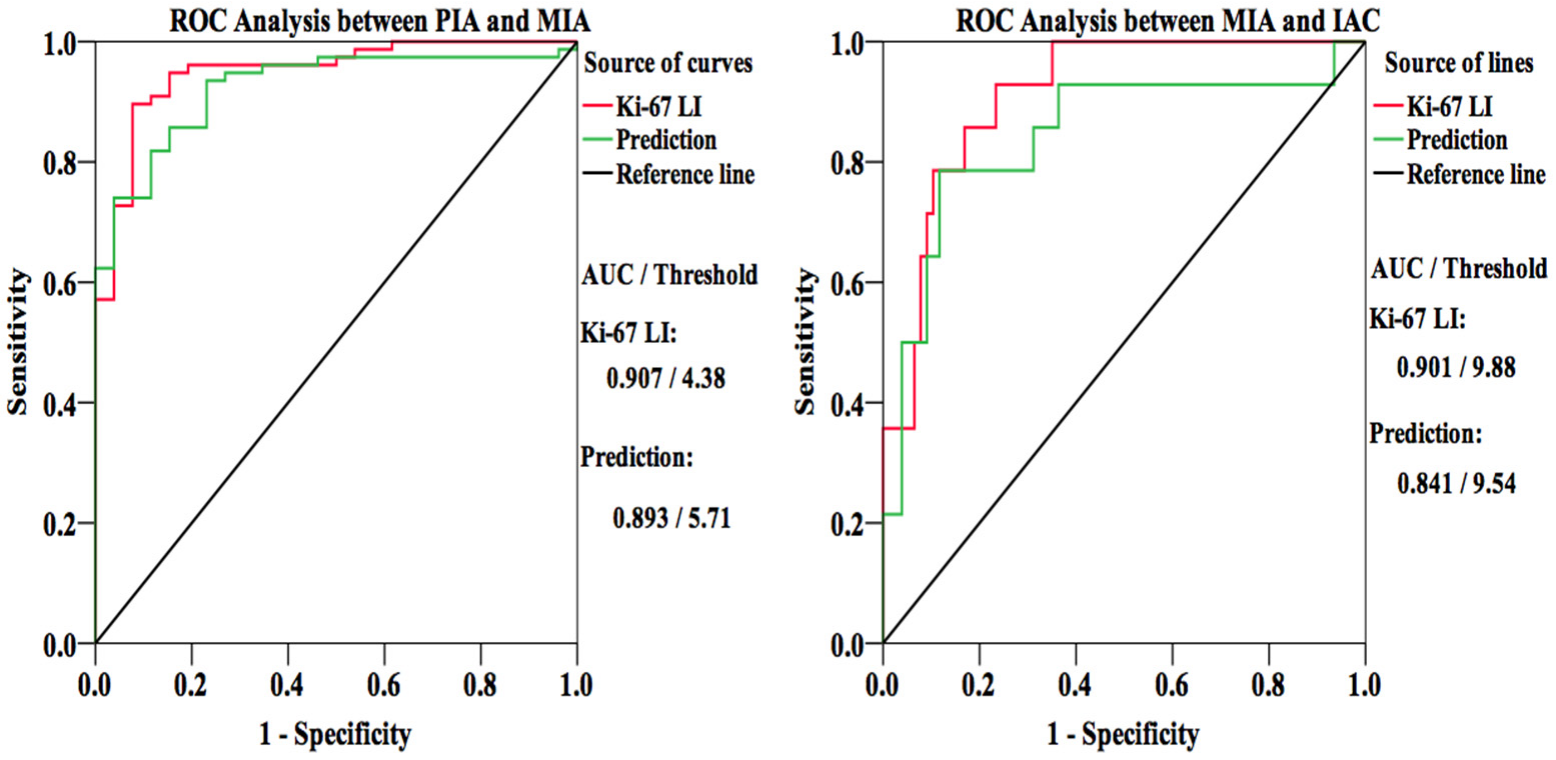
Receiver operating characteristic (ROC) curves of predictive Ki-67 labeling index (LI) and actual Ki-67 LI for comparison. Area under the curve (AUC) of predictive Ki-67 LI was lower than actual measured one, but approximated.

The prediction of Ki-67 LI was 4.27±0.76 (range, 2.67~6.05%), 6.50±1.69 (range,. 3.00~11.74%) and 8.81±3.73 (range, 3.75~16.65%) for PIA, MIA and IAC respectively (ANOVA P<0.001, LSD PIA vs. MIA P<0.001, MIA vs. IAC P = 0.014, PIA vs. IAC P<0.001). (Fig 7) Additionally, based on ROC analysis, we compared the AUC of the predictions with actual Ki-67 LI. Although discriminating not as well as actual Ki-67 LI does, the prediction of Ki-67 has a relatively higher AUC in ROC analysis to differentiate pathology of GGO nodules both between PIA and MIA (AUC = 0.893, Threshold = 5.71%) and between MIA and IAC (AUC = 0.841, Threshold = 9.54%) than diameter, TV, MAX, AVG and STD separately. (Fig 8)

## Discussion

Recent advances in imaging technology promoted the more precise and integrated 3D measurement of volume and CT number of pulmonary nodules than traditional one-dimensional or two-dimensional measurement methods.[15, 38–40] As for quantitative and more accurate analysis, given GGO lesion was not homogeneous and no clear dividing line exists between pure GGO and mix GGO, we just regarded them as a continuous progression paralleling to pathological progression from AAH through AIS to MIA. From this point of view, pure GGO is in a very beginning stage and mix GGO may be a middle one depending on percent of solid. Pure GGO lesions most likely own a low MAX, AVG and STD. When the first percent solid appear in pure GGO, it becomes “juvenile” mix GGO and own a higher MAX and little higher STD but a nearly AVG. Thereafter, solid component increased and moderate mix GGO comes up which own a larger diameter, MAX, AVG and STD.

Furthermore, although extensive studies have been conducted on the CT features of GGO nodules, little was known about the relationship between 3D parameters of GGO nodules and quantitative pathologic index such as the proliferative marker Ki-67. It is recorded that Ki-67 protein was originally defined by the prototype monoclonal antibody Ki-67[41], which was generated by immunizing mice with nuclei of the Hodgkin lymphoma cell line L428. The fact that the Ki-67 protein is present during all active phases of the cell cycle (G1, S, G2, and mitosis), but is absent from resting cells (G0), makes it an excellent marker for determining the so-called growth fraction of a given cell population[42]. High proliferative rate is a hallmark of cancer. Thus proliferation is reported to predict poor survival in multiple myeloma,[43] prostate cancer,[42, 44] and breast cancer.[45, 46]

However, on account of parameters such as size and CT number of GGO manifesting the quantity of cancerous cells, to a certain extent, as the same with Ki-67 LI in lung cancer, we considered that the correlation and regression model between them are biological and pathophysiological more than just statistical and mathematical. Most importantly, Ki-67 LIs in lesions presenting GGO of different pathological categories are significant difference. If we could predict it before surgery, radiologic diagnosis of GGO will be more accurate than only through radiological features. Therefore, preoperative prediction of Ki-67 LI could contribute to differential diagnosis of GGO nodules from not only radiology but pathology as well.

The pathological diagnosis is significant for selection of surgical procedures and prognosis. As a precursor of lung adenocarcinoma, malignant lesions undergo a progression along with AAH, AIS, MIA and IAC. As no invasion of lung parenchyma and lymph nodes, limited resections such as wedge resection and segmentectomy are appropriate for PIA including AAH and AIS. As for MIA and IAC that some cases have been detected invasion of local lymph nodes, standard lobectomy would be necessary.[47–50] AIS and MIA should have 100% and near 100% five-year disease-free survival (DFS) respectively when completely resected.[51–54]

In this study, diameter, TV, MAX, AVG, STD and Ki-67 LI were significantly and consecutively increasing along with the progression from PIA, MIA to IAC. Diameter and TV characterize GGO nodules from the perspective of whole basic framework of proliferation in comparison to MAX and AVG describing its substantiality and internality. Ultimately, STD manifests the degree of heterogeneity within the whole nodule. However, all of them are preoperative variables. Pathological diagnosis couldn’t be obtained before surgery or biopsy. Only if there is a quantitative pathologic index that could be measured or predicted preoperatively, can we accurately judge pathological features of GGO nodules.

By ROC analysis, the threshold of diameter and AVG for differentiating PIA from MIA and MIA from IAC was 10.55 mm, 21.8 mm, -615 HU, -464 HU respectively. Lee HY[55] explored the threshold of diameter and AVG of pure GGO for discriminating invasive adenocarcinoma with 15 mm and -472 HU respectively. By ROC analysis as well, Mamoru Takahashi[56] got the threshold of diameter for differentiating noninvasive lesions from invasive with 10 mm.

By Spearman correlative analysis, all of our radiologic image parameters have a significant positive correlation with Ki-67 LI. The increasing of Ki-67 LI along with the progression means rising proliferation of cancer cells. It made the parenchyma more thick and substantial, which present a higher CT number on HRCT. As for the invasion of MIA and IAC presenting prompt proliferation of cancerous cells at scattered point in lung tissue, thicknesses within lesion will be out of consistent step resulting in higher STD. Therefore, it is reasonable to presume that Ki-67 LI from pathology corresponds to parameters with regard to size and CT number from radiology. Furthermore, aim to predict Ki-67 LI preoperatively, we established a multivariable linear regression equation to assess proliferative degree of GGO nodules approximately and compared the prediction Ki-67 LI with actual one. AUC and threshold of predictive Ki-67 LI were not only close to those of actual one, but also better than those of other parameters in this study separately. We obtained the predictable equation as follow: Prediction (Ki-67 LI) = 0.022*STD+0.001* TV+2.137.

Some papers had shown that DNA synthesis was inhibited by oligodeoxynucleotides complementary to Ki-67 mRNA. Moreover, the microinjection of antibodies directed against the murine Ki-67 or the human Ki-67 resulted in a decreased rate of cell division. The phosphorylation and dephosphorylation of the Ki-67 protein controlled by the key regulatory complex cyclin B/cdc 2 is parallel to the transit of cells through mitosis.[57–60] As for volume of lesion in the equation, benign lung parenchyma around fringe of lesion would proliferate when Ki-67 LI increased. The pixels there then would get higher density distinguishing parenchyma around nodule than more peripheral structure and making them become part of GGO lesion. It is obvious and understandable that volume of GGO would increase along with Ki-67 LI. STD means the degree of heterogeneity of nodule. (Fig 1) When a lesion appeared, it presented pure GGO judged by subjective observation. In AAH, cells all over the nodule just proliferate with a low expression of Ki-67 so that difference of proliferating capability among cells was small, resulting in low STD on the CT screen. In AIS, proliferation was constrained in alveolar epithelium where have a higher CT number. Interstitial tissue was clean and has not been invaded by carcinoma cells. So it presented lower CT number. Then STD increased due to increasing difference of CT numbers among scattered proliferating areas. As lung adenocarcinoma progressing, in MIA or IAC, more cells express Ki-67 and proliferate irregularly. Some fast growing areas in nodule produced solid and other slow ones remained ground glass attenuation. Invasive growing made some interstitium filled with proliferating carcinoma cells. Though, in the solid part, pixels would not manifest the same density in CT screen. In short, higher Ki-67 LI, more irregular proliferation and larger STD would accordingly appear along with progression of lung adenocarcinoma. Just as result of Spearman correlative analysis, TV and STD had a significant positive correlation with Ki-67LI.

In conclusion, image parameters, such as diameter, TV, MAX, AVG and STD based on 3D CT, could discriminate the different pathology in the progression of lung adenocarcinoma and contribute to prognosis relatively as well. Additionally, we established a regression formula to predict Ki-67 labeling index of GGO lesions based on those parameters so that we can estimate the GGO pathologic status more accuracy preoperatively.
