# Correction: Preoperative Prediction of Ki-67 Labeling Index By Three-dimensional CT Image Parameters for Differential Diagnosis Of Ground-Glass Opacity (GGO)

**DOI:** 10.1371/journal.pone.0211950

**Published:** 2019-02-01

**Authors:** Mingzheng Peng, Fei Peng, Chengzhong Zhang, Qingguo Wang, Zhao Li, Haiyang Hu, Sida Liu, Binbin Xu, Wenzhuo Zhu, Yudong Han, Qiang Lin

The Data Availability statement for this paper is incorrect. The correct statement is: The authors confirm that all data underlying the findings are fully available without restriction. All relevant data are within the paper and its Supporting Information files.

S1 File is omitted from the list of Supporting Information. It can be viewed below.

## Supporting information

S1 File Supplementary dataset. This file includes supplementary data.
(ZIP)
