# Supplementary material for: Correction: Preoperative Prediction of Ki-67 Labeling Index By Three-dimensional CT Image Parameters for Differential Diagnosis Of Ground-Glass Opacity (GGO)
Source: PLoS One. 2019 Feb 1;14(2):e0211950. doi: 10.1371/journal.pone.0211950 (PMC6358103; doi:10.1371/journal.pone.0211950)
Supplement: S1 File — This file includes supplementary data. (ZIP) [file pone.0211950.s001.zip › statistical analyses.doc]

GET DATA /TYPE=XLSX
  /FILE='/Users/pengmingzheng/Documents/ggo ct/ materials and data.xlsx'
  /SHEET=name 'Raw-materials'
  /CELLRANGE=full
  /READNAMES=on
  /ASSUMEDSTRWIDTH=32767.
EXECUTE.
DATASET NAME DATA1 WINDOW=FRONT.
REGRESSION
  /DESCRIPTIVES MEAN STDDEV CORR SIG N
  /MISSING LISTWISE
  /STATISTICS COEFF OUTS CI(95) R ANOVA
  /CRITERIA=PIN(.05) POUT(.10)
  /NOORIGIN
  /DEPENDENT ki67
  /METHOD=ENTER Diameter TV.

One-way


One-way analysis of variance 	
	Sum of squares	df	Mean square	F	Sig.	
Diameter	组é间ä	1163.926	2	581.963	20.354	.000	
	组é内Ú	2973.609	104	28.592			
	总Ü数ý	4137.535	106				
TV	组é间ä	16102001.996	2	8051000.998	12.242	.000	
	组é内Ú	68398492.883	104	657677.816			
	总Ü数ý	84500494.879	106				

GET DATA /TYPE=XLSX
  /FILE='/Users/pengmingzheng/Documents/ggo ct/materials and data.xlsx'
  /SHEET=name 'Raw-materials'
  /CELLRANGE=full
  /READNAMES=on
  /ASSUMEDSTRWIDTH=32767.
EXECUTE.
DATASET NAME DATA4 WINDOW=FRONT.

SAVE OUTFILE='/Users/pengmingzheng/Documents/ggo ct/materials and data.sav'
  /COMPRESSED.
MEANS TABLES=age BY PPP
  /CELLS MEAN COUNT STDDEV
  /STATISTICS ANOVA.


Mean


Annotations	
Output	30-JAN-2015 13:16:20	
Annotations		
Input	DATA	/Users/pengmingzheng/Documents/ggo ct/materials and data.sav	
	Active data	DATA4	
	Filger	<none>	
	Weight	<none>	
	Files	<none>	
		160	

[DATA4] /Users/pengmingzheng/Documents/ggo ct/materials and data.sav


Case processing summary	
	Cases	
	Included	Excluded	Total	
	N	Percentage	N	Percentage	N	Percentage	
age  * PPP	160	100.0%	0	0.0%	160	100.0%	


Report	
age	
PPP	Mean	N	Std	
1	53.05	37	8.120	
2	56.69	106	10.276	
3	62.94	17	7.570	
总Ü计Æ	56.51	160	9.882	


ANOVA 	
	Sum of squares	df	MS	F	Sig.	
age * PPP	Inter-group	1148.416	2	574.208	6.270	.002	
	Intra-group	14377.559	157	91.577			
	Total	15525.975	159				


Correlation	
	Eta	Eta square	
age * PPP	.272	.074	

MEANS TABLES=MAX AVG STD Ki67# CVprediction Diameter TV BY PPP_B
  /CELLS MEAN COUNT STDDEV
  /STATISTICS ANOVA.


Mean

 [DATA4] /Users/pengmingzheng/Documents/ggo ct/materials and data.sav


Case processing summary	
	Cases	
	Included	Excluded	Total	
	N	Percentage	N	Percentage	N	Percentage	
MAX  * PPP	117	73.1%	43	26.9%	160	100.0%	
AVG  * PPP	117	73.1%	43	26.9%	160	100.0%	
STD  * PPP	117	73.1%	43	26.9%	160	100.0%	
Ki-67#  * PPP	113	70.6%	47	29.4%	160	100.0%	
CV-prediction  * PPP	117	73.1%	43	26.9%	160	100.0%	
Diameter  * PPP	117	73.1%	43	26.9%	160	100.0%	
TV  * PPP	117	73.1%	43	26.9%	160	100.0%	


Report	
PPP	MAX	AVG	STD	Ki-67#	CV-prediction	Diameter	TV	
1.000	Mean	-281.1538462	-656.6153846	88.3000000	2.7941692	4.3233308	9.06923	197.23077	
	N	26	26	26	26	26	26	26	
	Std	182.54088688	48.25936338	28.65147815	1.31906991	.82137051	3.701565	199.968259	
2.000	Mean	21.0909091	-575.8311688	155.1272727	6.2606130	6.4939818	15.61347	959.88312	
	N	77	77	77	73	77	77	77	
	Std	182.20297977	56.03931951	41.82743571	1.66444280	1.70341214	7.191157	1148.498791	
3.000	Mean	230.3571429	-516.4285714	182.0214286	9.7154571	8.8139714	23.85721	2672.50000	
	N	14	14	14	14	14	14	14	
	Std	397.06240686	105.74040366	62.69929675	2.23518381	3.72689592	9.320805	2572.434039	
总Ü计Æ	Mean	-21.0341880	-586.6752137	143.4948718	5.8910580	6.2892205	15.14562	995.33333	
	N	117	117	117	113	117	117	117	
	Std	265.65235033	74.71610973	52.11066283	2.62543648	2.29487422	8.009391	1448.281449	


ANOVA 	
	SS	df	MS	F	Sig	
MAX * PPP	Inter-group	2780622.901	2	1390311.450	29.320	.000	
	Intra-group	5405632.963	114	47417.833			
	Total	8186255.863	116				
AVG * PPP	Inter-group	205321.271	2	102660.635	26.463	.000	
	Intra-group	442248.388	114	3879.372			
	Total	647569.658	116				
STD * PPP	Inter-group	110407.541	2	55203.770	30.760	.000	
	Intra-group	204592.916	114	1794.675			
	Total	315000.457	116				
Ki-67# * PPP	Inter-group	464.093	2	232.046	82.897	.000	
	Intra-group	307.914	110	2.799			
	Total	772.007	112				
CV-prediction * PPP	Inter-group	192.952	2	96.476	26.314	.000	
	Intra-group	417.956	114	3.666			
	Total	610.908	116				
Diameter * PPP	Inter-group	2039.325	2	1019.663	21.518	.000	
	Intra-group	5402.114	114	47.387			
	Total	7441.439	116				
TV * PPP	Inter-group	56038359.937	2	28019179.968	17.056	.000	
	Intra-group	187273862.063	114	1642753.176			
	Total	243312222.000	116				


Correlation	
	Eta	Eta square	
MAX * PPP	.583	.340	
AVG * PPP	.563	.317	
STD * PPP	.592	.350	
Ki-67# * PPP	.775	.601	
CV-prediction * PPP	.562	.316	
Diameter * PPP	.523	.274	
TV * PPP	.480	.230	

NONPAR CORR
  /VARIABLES=MAX Diameter AVG STD TV ki67#
  /PRINT=BOTH TWOTAIL NOSIG
  /MISSING=PAIRWISE.


Nonparametric correlation coefficient


 [DATA4] 


Correlation coefficient	
	MAX	Diameter	AVG	STD	TV	ki-67(%)	
Kendall's tau_b	MAX	CC	1.000	.625	.581	.657	.582	.426	
		Sig.	.	.000	.000	.000	.000	.000	
		N	129	129	129	129	129	129	
	Diameter	CC	.625	1.000	.441	.499	.854	.412	
		Sig	.000	.	.000	.000	.000	.000	
		N	129	129	129	129	129	129	
	AVG	CC	.581	.441	1.000	.662	.438	.406	
		Sig.	.000	.000	.	.000	.000	.000	
		N	129	129	129	129	129	129	
	STD	CC	.657	.499	.662	1.000	.459	.465	
		Sig.	.000	.000	.000	.	.000	.000	
		N	129	129	129	129	129	129	
	TV	CC	.582	.854	.438	.459	1.000	.395	
		Sig.	.000	.000	.000	.000	.	.000	
		N	129	129	129	129	129	129	
	ki-67(%)	CC	.426	.412	.406	.465	.395	1.000	
		Sig.	.000	.000	.000	.000	.000	.	
		N	129	129	129	129	129	129	
Spearman's rho	MAX	CC	1.000	.812	.777	.834	.773	.601	
		Sig.	.	.000	.000	.000	.000	.000	
		N	129	129	129	129	129	129	
	Diameter	CC	.812	1.000	.603	.675	.964	.575	
		Sig.	.000	.	.000	.000	.000	.000	
		N	129	129	129	129	129	129	
	AVG	CC	.777	.603	1.000	.847	.604	.585	
		Sig.	.000	.000	.	.000	.000	.000	
		N	129	129	129	129	129	129	
	STD	CC	.834	.675	.847	1.000	.628	.639	
		Sig.	.000	.000	.000	.	.000	.000	
		N	129	129	129	129	129	129	
	TV	CC	.773	.964	.604	.628	1.000	.559	
		Sig.	.000	.000	.000	.000	.	.000	
		N	129	129	129	129	129	129	
	ki-67(%)	CC	.601	.575	.585	.639	.559	1.000	
		Sig.	.000	.000	.000	.000	.000	.	
		N	129	129	129	129	129	129	

GRAPH
  /SCATTERPLOT(BIVAR)=ki67# WITH STD
  /MISSING=LISTWISE.


Chart


[DATA4] 


GRAPH
  /SCATTERPLOT(BIVAR)=ki67# WITH AVG
  /MISSING=LISTWISE.


Chart


[DATA4] 


GRAPH
  /SCATTERPLOT(BIVAR)=ki67# WITH MAX
  /MISSING=LISTWISE.


Chart


[DATA4] 


GRAPH
  /SCATTERPLOT(BIVAR)=ki67# WITH Diameter
  /MISSING=LISTWISE.


Chart

 [DATA4] 


GRAPH
  /SCATTERPLOT(BIVAR)=ki67# WITH TV
  /MISSING=LISTWISE.


Chart


[DATA4] 


EXAMINE VARIABLES=STD BY PPP
  /PLOT=BOXPLOT
  /STATISTICS=NONE
  /NOTOTAL.


[DATA11] 


PPP


Cases processing summary	
	PPP	Cases	
		Effective	Missing	Total	
		N	Percentage	N	Percentage	N	Percentage	
STD	PIA(AAH+AIS)	26	100.0%	0	0.0%	26	100.0%	
	MIA	77	100.0%	0	0.0%	77	100.0%	
	IAC	14	100.0%	0	0.0%	14	100.0%	


STD


EXAMINE VARIABLES=AVG BY PPP
  /PLOT=BOXPLOT
  /STATISTICS=NONE
  /NOTOTAL.


[DATA11] 


PPP


Cases processing summary	
	PPP	Cases	
		Effective	Missing	Total	
		N	Percentage	N	Percentage	N	Percentage	
AVG	PIA(AAH+AIS)	26	100.0%	0	0.0%	26	100.0%	
	MIA	77	100.0%	0	0.0%	77	100.0%	
	IAC	14	100.0%	0	0.0%	14	100.0%	


AVG


EXAMINE VARIABLES=MAX BY PPP
  /PLOT=BOXPLOT
  /STATISTICS=NONE
  /NOTOTAL.


[DATA11] 


PPP


Cases processing summary	
	PPP	Cases	
		Effective	Missing	Total	
		N	Percentage	N	Percentage	N	Percentage	
MAX	PIA(AAH+AIS)	26	100.0%	0	0.0%	26	100.0%	
	MIA	77	100.0%	0	0.0%	77	100.0%	
	IAC	14	100.0%	0	0.0%	14	100.0%	


MAX


EXAMINE VARIABLES=ki67# BY PPP
  /PLOT=BOXPLOT
  /STATISTICS=NONE
  /NOTOTAL.


[DATA11] 


PPP


Cases processing summary	
	PPP	Cases	
		Effective	Missing	Total	
		N	Percentage	N	Percentage	N	Percentage	
ki-67(%)	PIA(AAH+AIS)	26	100.0%	0	0.0%	26	100.0%	
	MIA	77	100.0%	0	0.0%	77	100.0%	
	IAC	14	100.0%	0	0.0%	14	100.0%	


ki-67(%)


EXAMINE VARIABLES=Diameter BY PPP
  /PLOT=BOXPLOT
  /STATISTICS=NONE
  /NOTOTAL.


[DATA11] 


PPP


Cases processing summary	
	PPP	Cases	
		Effective	Missing	Total	
		N	Percentage	N	Percentage	N	Percentage	
Diameter	PIA(AAH+AIS)	26	100.0%	0	0.0%	26	100.0%	
	MIA	77	100.0%	0	0.0%	77	100.0%	
	IAC	14	100.0%	0	0.0%	14	100.0%	


Diameter


GRAPH
  /SCATTERPLOT(BIVAR)=C.solid WITH STD
  /MISSING=LISTWISE.


[DATA1] 


EXAMINE VARIABLES=TV BY PPP
  /PLOT=BOXPLOT
  /STATISTICS=NONE
  /NOTOTAL.


[DATA11] 


PPP


Cases processing summary	
	PPP	Cases	
		Effective	Missing	Total	
		N	Percentage	N	Percentage	N	Percentage	
TV	PIA(AAH+AIS)	26	100.0%	0	0.0%	26	100.0%	
	MIA	77	100.0%	0	0.0%	77	100.0%	
	IAC	14	100.0%	0	0.0%	14	100.0%	


TV


EXAMINE VARIABLES=CVprediction BY PPP
  /PLOT=BOXPLOT
  /STATISTICS=NONE
  /NOTOTAL.


[DATA1] 


PPP


Cases processing summary	
	PPP	Cases	
		Effective	Missing	Total	
		N	Percentage	N	Percentage	N	Percentage	
CV-prediction	1.00000	26	100.0%	0	0.0%	26	100.0%	
	2.00000	77	100.0%	0	0.0%	77	100.0%	
	3.00000	14	100.0%	0	0.0%	14	100.0%	


CV-prediction


USE ALL.
COMPUTE filter_$=(PPP=2 OR PPP=1).
VARIABLE LABELS filter_$ 'PPP=2 OR PPP=1 (FILTER)'.
VALUE LABELS filter_$ 0 'Not Selected' 1 'Selected'.
FORMATS filter_$ (f1.0).
FILTER BY filter_$.
EXECUTE.
ROC Diameter TV MAX AVG STD ki67 BY PPP (2)
  /PLOT=CURVE(REFERENCE)
  /PRINT=SE
  /CRITERIA=CUTOFF(INCLUDE) TESTPOS(LARGE) DISTRIBUTION(FREE) CI(95)
  /MISSING=EXCLUDE.


ROC Curve


[DATA6] 


Cases processing summary	
PPP	Effective N	
Positive	77	
Negtive	26	


Area under the curve (AUC)	
Variables	Area	Std. error	 Sig.	 95% confidence Interval	
				Lower limit	Upper limit	
Diameter	.801	.048	.000	.708	.895	
TV	.822	.043	.000	.737	.907	
MAX	.890	.037	.000	.817	.963	
AVG	.857	.042	.000	.774	.939	
STD	.901	.030	.000	.843	.959	
ki-67	.907	.023	.000	.905	.994	

USE ALL.
COMPUTE filter_$=(PPP=3 or PPP=2).
VARIABLE LABELS filter_$ 'PPP=3 or PPP=2 (FILTER)'.
VALUE LABELS filter_$ 0 'Not Selected' 1 'Selected'.
FORMATS filter_$ (f1.0).
FILTER BY filter_$.
EXECUTE.
ROC Diameter TV MAX AVG STD ki67 BY PPP (3)
  /PLOT=CURVE(REFERENCE)
  /PRINT=SE COORDINATES
  /CRITERIA=CUTOFF(INCLUDE) TESTPOS(LARGE) DISTRIBUTION(FREE) CI(95)
  /MISSING=EXCLUDE.


GET DATA /TYPE=XLSX
  /FILE='C:\Documents and Settings\Administrator\materials and data.xlsx'
  /SHEET=name 'Raw-materials'
  /CELLRANGE=full
  /READNAMES=on
  /ASSUMEDSTRWIDTH=32767.
EXECUTE.
DATASET NAME DATA1 WINDOW=FRONT.
REGRESSION
  /DESCRIPTIVES MEAN STDDEV CORR SIG N
  /MISSING LISTWISE
  /STATISTICS COEFF OUTS R ANOVA CHANGE
  /CRITERIA=PIN(.05) POUT(.10)
  /NOORIGIN
  /DEPENDENT ki67
  /METHOD=STEPWISE Diameter TV MAX AVG STD.


Regression


[DATA1] 


Descriptive statistics	
	Mean	Std.	N	
ki-67	5.7606679	2.67300542	117	
Diameter	15.145584097	8.0093906525	117	
TV	995.3333333	1448.28144888	117	
MAX	-21.0341880	265.65235033	117	
AVG	-586.6752137	74.71610973	117	
STD	143.4948718	52.11066283	117	


Correlation	
	ki-67	Diameter	TV	MAX	AVG	STD	
Pearson correlation	ki-67	1.000	.504	.483	.494	.468	.533	
	Diameter	.504	1.000	.886	.732	.621	.677	
	TV	.483	.886	1.000	.639	.553	.558	
	MAX	.494	.732	.639	1.000	.757	.757	
	AVG	.468	.621	.553	.757	1.000	.838	
	STD	.533	.677	.558	.757	.838	1.000	
Sig. 	ki-67	.	.000	.000	.000	.000	.000	
	Diameter	.000	.	.000	.000	.000	.000	
	TV	.000	.000	.	.000	.000	.000	
	MAX	.000	.000	.000	.	.000	.000	
	AVG	.000	.000	.000	.000	.	.000	
	STD	.000	.000	.000	.000	.000	.	
N	ki-67	117	117	117	117	117	117	
	Diameter	117	117	117	117	117	117	
	TV	117	117	117	117	117	117	
	MAX	117	117	117	117	117	117	
	AVG	117	117	117	117	117	117	
	STD	117	117	117	117	117	117	


Inputted／¯removed variablesa	
Model	Inputted variables	Removed varbiables	Methods	
1	STD	.	Progress（¨rules: probability of F-to-enter  <= .050，¬F-to-remove probability of >= .100）©。£	
2	TV	.	Progress（¨rul: probability of F-to-enter <= .050，¬probability of F-to-remove >= .100）©。£	


Model summary	
Models	R	R square	adjusted R square	Standard estimated error 	Changed statistics	
					R square change	F change	df1	df2	Sig. F change	
1	.533a	.284	.278	2.27199758	.284	45.562	1	115	.000	
2	.578b	.334	.322	2.20074557	.050	8.567	1	114	.004	


Anovac	
Models	Sum of square	df	Mean square	F	Sig.	
1	Regression	235.188	1	235.188	45.562	.000a	
	Residual	593.627	115	5.162			
	Total	828.815	116				
2	Regression	276.681	2	138.341	28.563	.000b	
	Residual	552.134	114	4.843			
	Total	828.815	116				


coffecienta	
Models	Non-standardized coefficient 	Standardized coefficient	t	Sig.	
	B	Std. error	Trail version			
1	(Constant)	1.840	.618		2.978	.004	
	STD	.027	.004	.533	6.750	.000	
2	(Constant)	2.453	.634		3.869	.000	
	STD	.020	.005	.382	4.146	.000	
	TV	.000	.000	.270	2.927	.004	


Excluded variablesc	
Models	Beta In	t	Sig.	Partial correlation	Collinear statistics	
					Tolerance	
1	Diameter	.265a	2.525	.013	.230	.542	
	TV	.270a	2.927	.004	.264	.688	
	MAX	.213a	1.779	.078	.164	.428	
	AVG	.074a	.507	.613	.047	.298	
2	Diameter	.038b	.203	.840	.019	.167	
	MAX	.091b	.710	.479	.067	.360	
	AVG	-.004b	-.026	.979	-.002	.287	

REGRESSION
  /MISSING LISTWISE
  /STATISTICS COEFF OUTS R ANOVA
  /CRITERIA=PIN(.05) POUT(.10)
  /NOORIGIN
  /DEPENDENT ki67
  /METHOD=STEPWISE Diameter TV MAX AVG STD.


Regression


[DATA2] 

Inputted／¯removed variablesa	
Model	Inputted variables	Removed varbiables	Methods	
1	STD	.	Progress（¨rules: probability of F-to-enter  <= .050，¬F-to-remove probability of >= .100）©。£	
2	TV	.	Progress（¨rul: probability of F-to-enter <= .050，¬probability of F-to-remove >= .100）©。£	


Model summary	
Models	R	R square	adjusted R square	Standardized estimated error 	
1	.533	.284	.278	2.271990	
2	.578	.334	.322	2.200740	


Anova	
Models	Sum of squares	df	均ù方½	F	Sig.	
1	Regression	235.192	1	235.192	45.563	.000	
	Residual	593.623	115	5.162			
	Total	828.815	116				
2	Regression	276.684	2	138.342	28.564	.000	
	Residual	552.131	114	4.843			
	Total	828.815	116				


Coefficient	
Models	Non-standardized coefficient	Standardized coefficient	t	Sig.	
	B	Std. error	Trail version			
1	(Constant)	1.840	.618		2.978	.004	
	STD	.027	.004	.533	6.750	.000	
2	(Constant)	2.453	.634		3.869	.000	
	STD	.020	.005	.382	4.146	.000	
	TV	.000	.000	.270	2.927	.004	


Excluded variables	
Models	Beta In	t	Sig.	Partial correlation	Collinear statistics	
					Tolerance	
1	Diameter	.265	2.525	.013	.230	.542	
	TV	.270	2.927	.004	.264	.688	
	MAX	.213	1.779	.078	.164	.428	
	AVG	.074	.507	.613	.047	.298	
2	Diameter	.038	.203	.840	.019	.167	
	MAX	.091	.709	.480	.067	.360	
	AVG	-.004	-.026	.979	-.002	.287	

REGRESSION
  /MISSING LISTWISE
  /STATISTICS COEFF OUTS R ANOVA
  /CRITERIA=PIN(.05) POUT(.10)
  /NOORIGIN
  /DEPENDENT ki67
  /METHOD=BACKWARD Diameter TV MAX AVG STD.


Regression


[DATA2] 


Inputted/removed variables 	
Models	Inputted variables	Removed variables	Methods	
1	STD, TV, MAX, AVG, Diameter	.	Input	
2	.	Diameter	backward（¨rule: F-to-remove >= .100）©.	
3	.	AVG	backward（¨rule: F-to-remove >= .100）©.	
4	.	MAX	backward（¨rule: F-to-remove >= .100）©.	


Model summary	
Models	R	R square	adjusted R square	Standardized estimated error	
1	.581	.337	.307	2.224716	
2	.581	.337	.313	2.214762	
3	.580	.337	.319	2.205550	
4	.578	.334	.322	2.200740	


Anova	
Models	Sum of suqare	df	Mean square	F	Sig.	
1	Regression	279.436	5	55.887	11.292	.000	
	Residual	549.379	111	4.949			
	Total	828.815	116				
2	Regression	279.436	4	69.859	14.242	.000	
	Residual	549.379	112	4.905			
	Total	828.815	116				
3	Regression	279.132	3	93.044	19.127	.000	
	Residual	549.683	113	4.864			
	Total	828.815	116				
4	Regression	276.684	2	138.342	28.564	.000	
	Residual	552.131	114	4.843			
	Total	828.815	116				


Coefficient	
Models	非Ç标ê准¼化¯系µ数ý	标ê准¼系µ数ý	t	Sig.	
	B	标ê准¼ 误ó差î	试Ô用Ã版æ			
1	(constant)	1.956	3.965		.493	.623	
	Diameter	.000	.066	.000	-.002	.998	
	TV	.000	.000	.243	1.434	.154	
	MAX	.001	.001	.101	.718	.474	
	AVG	-.001	.005	-.037	-.246	.806	
	STD	.018	.008	.352	2.235	.027	
2	(constant)	1.956	3.947		.496	.621	
	TV	.000	.000	.243	2.396	.018	
	MAX	.001	.001	.101	.749	.456	
	AVG	-.001	.005	-.037	-.249	.804	
	STD	.018	.008	.352	2.339	.021	
3	(constant)	2.912	.907		3.211	.002	
	TV	.000	.000	.241	2.395	.018	
	MAX	.001	.001	.091	.709	.480	
	STD	.017	.006	.329	2.780	.006	
4	(constant)	2.453	.634		3.869	.000	
	TV	.000	.000	.270	2.927	.004	
	STD	.020	.005	.382	4.146	.000	


Excluded variables	
Modoles	Beta In	t	Sig.	Partial correlation	Collinear statistics	
					Tolerance	
2	Diameter	.000	-.002	.998	.000	.154	
3	Diameter	.006	.029	.977	.003	.157	
	AVG	-.037	-.249	.804	-.024	.261	
4	Diameter	.038	.203	.840	.019	.167	
	AVG	-.004	-.026	.979	-.002	.287	
	MAX	.091	.709	.480	.067	.360	

*Nonparametric Tests: Independent Samples.
NPTESTS
  /INDEPENDENT TEST (Diameter TV MAX AVG STD ki67# CVprediction) GROUP (PPP)
  /MISSING SCOPE=ANALYSIS USERMISSING=EXCLUDE
  /CRITERIA ALPHA=0.05  CILEVEL=95.


non-parametric test


[DATA1] 


NPAR TESTS
  /K-W=Diameter TV MAX AVG STD ki67# Prediction原­）© BY PPP(1 3)
  /STATISTICS DESCRIPTIVES
  /MISSING ANALYSIS.


NPar test


[DATA1] 


Descriptive statistics	
	N	Mean	Std. 	Min	Max	
Diameter	117	15.1455844	8.00939056	3.26667	43.20000	
TV	117	995.3333333	1448.28144888	17.00000	7979.00000	
MAX	117	-21.0341880	265.65235033	-600.00000	1160.00000	
AVG	117	-586.6752137	74.71610973	-757.00000	-366.00000	
STD	117	143.4948718	52.11066283	23.40000	301.50000	
ki-67#	117	5.7606679	2.67300542	1.29200	15.00000	
Prediction(原­）©	117	5.0927650	1.86406317	2.82000	13.46500	
PPP	117	1.8974359	.57811548	1.00000	3.00000	


Kruskal-Wallis test


Rank	
	PPP	N	Mean of rank	
Diameter	1.00000	26	29.56	
	2.00000	77	63.12	
	3.00000	14	91.04	
	Total	117		
TV	1.00000	26	27.88	
	2.00000	77	63.90	
	3.00000	14	89.86	
	Total	117		
MAX	1.00000	26	23.33	
	2.00000	77	66.31	
	3.00000	14	85.04	
	Total	117		
AVG	1.00000	26	26.19	
	2.00000	77	65.62	
	3.00000	14	83.54	
	Total	117		
STD	1.00000	26	22.35	
	2.00000	77	67.31	
	3.00000	14	81.36	
	Total	117		
ki-67#	1.00000	26	17.38	
	2.00000	77	64.92	
	3.00000	14	103.71	
	Total	117		
Prediction(原­）©	1.00000	26	22.81	
	2.00000	77	66.16	
	3.00000	14	86.86	
	Total	117		


Test statistics	
	Diameter	TV	MAX	AVG	STD	ki-67#	Prediction	
Chi-square	33.215	35.072	40.588	34.583	41.069	65.815	42.473	
df	2	2	2	2	2	2	2	
Progressive sig.	.000	.000	.000	.000	.000	.000	.000	

EXAMINE VARIABLES=CVprediction BY PPP
  /PLOT=BOXPLOT
  /STATISTICS=NONE
  /NOTOTAL.


skim


[DATA1] 


PPP


Case processing summary	
	PPP	Cases	
		Effective	Missing	Total	
		N	Percentage	N	Percentage	N	Percentage	
CV-prediction	1.00000	26	100.0%	0	0.0%	26	100.0%	
	2.00000	77	100.0%	0	0.0%	77	100.0%	
	3.00000	14	100.0%	0	0.0%	14	100.0%	


CV-prediction


FILTER OFF.
USE ALL.
EXECUTE.
GRAPH
  /SCATTERPLOT(MATRIX)=ki67 predicted STD
  /MISSING=LISTWISE.


Chart


[DATA2] 


ROC Prediction ki67# BY PPP (3)
  /PLOT=CURVE(REFERENCE)
  /PRINT=SE COORDINATES
  /CRITERIA=CUTOFF(INCLUDE) TESTPOS(LARGE) DISTRIBUTION(FREE) CI(95)
  /MISSING=EXCLUDE.


ROC Curve

[DATA2] /Users/pengmingzheng/Documents/ggo ct/materials and data.sav


Case processing summary	
PPP	effective N	
Positive	14	
Negative	77	


Area under the curve (AUC)	
Test variables	Area	Std. error	Progressive Sig.	 95% confidence interval	
				Lower limit	Upper limit	
Prediction	.841	.068	.000	.712	.977	
ki-67#	.902	.034	.000	.846	.978	


Coordinate of the curve	
Test variables	Positive if ≥Ý	Sensitivity	1 －­ specificity	
Prediction	.0400000	1.000	1.000	
	1.9300000	1.000	.987	
	3.1487500	1.000	.974	
	3.5955000	1.000	.961	
	3.7820000	1.000	.948	
	3.8605000	1.000	.935	
	3.8827500	.929	.935	
	3.9195000	.929	.922	
	3.9520000	.929	.909	
	3.9607500	.929	.896	
	3.9685000	.929	.883	
	3.9962500	.929	.870	
	4.0307500	.929	.857	
	4.0460000	.929	.844	
	4.0512500	.929	.831	
	4.0665000	.929	.818	
	4.1007500	.929	.792	
	4.1332500	.929	.779	
	4.1467500	.929	.766	
	4.1510000	.929	.753	
	4.2412500	.929	.740	
	4.3385000	.929	.727	
	4.3765000	.929	.714	
	4.4130000	.929	.701	
	4.4397500	.929	.688	
	4.4632500	.929	.675	
	4.4817500	.929	.662	
	4.5402500	.929	.649	
	4.5950000	.929	.636	
	4.6357500	.929	.623	
	4.6665000	.929	.610	
	4.6995000	.929	.584	
	4.7415000	.929	.571	
	4.7632500	.929	.558	
	4.7932500	.929	.545	
	4.8315000	.929	.532	
	4.8527500	.929	.519	
	4.8557500	.929	.506	
	4.8640000	.929	.494	
	4.8750000	.929	.481	
	4.8862500	.929	.468	
	4.9110000	.929	.455	
	4.9295000	.929	.442	
	4.9377500	.929	.429	
	4.9495000	.929	.416	
	5.0025000	.929	.403	

Coordinates of the curve	
Test variables	Positive if ≥Ý	Sensitivity	1 －­ specificity	
Prediction	5.0575000	.929	.377	
	5.1272500	.929	.364	
	5.2140000	.857	.364	
	5.2660000	.857	.351	
	5.3265000	.857	.338	
	5.3747500	.857	.325	
	5.4052500	.857	.312	
	5.4209500	.786	.312	
	5.4394500	.786	.299	
	5.4675000	.786	.286	
	5.4892500	.786	.273	
	5.5032500	.786	.260	
	5.5170000	.786	.247	
	5.5417500	.786	.234	
	5.5587500	.786	.221	
	5.6237500	.786	.208	
	5.7142500	.786	.195	
	5.7737500	.786	.182	
	5.9680000	.786	.169	
	6.1572500	.786	.156	
	6.1997500	.786	.143	
	6.2515000	.786	.130	
	6.3710000	.786	.117	
	6.5805000	.714	.117	
	6.7202500	.643	.117	
	6.8665000	.643	.104	
	7.0802500	.643	.091	
	7.4582500	.571	.091	
	7.7637500	.500	.091	
	7.9535000	.500	.078	
	8.1382500	.500	.065	
	8.3535000	.500	.052	
	8.7002500	.500	.039	
	8.8895000	.429	.039	
	8.9830000	.357	.039	
	9.2255000	.286	.039	
	9.6550000	.214	.039	
	9.9380000	.214	.026	
	10.2270000	.214	.013	
	11.0215000	.214	.000	
	12.1555000	.143	.000	
	13.0967500	.071	.000	
	14.4650000	.000	.000	
ki-67#	.9046000	1.000	1.000	
	2.1688000	1.000	.987	
	2.4440000	1.000	.974	

Coordinates of the curve	
Test variables	Positive if ≥Ý	Sensitivity	1 －­ specificity	
ki-67#	2.9609500	1.000	.961	
	3.6809500	1.000	.948	
	3.9285000	1.000	.935	
	4.0032500	1.000	.922	
	4.1961000	1.000	.909	
	4.3664000	1.000	.896	
	4.4391000	1.000	.883	
	4.5085500	1.000	.870	
	4.5370000	1.000	.857	
	4.5890000	1.000	.844	
	4.6462500	1.000	.831	
	4.7192500	1.000	.818	
	4.8262500	1.000	.805	
	4.8862500	1.000	.792	
	4.9720000	1.000	.779	
	5.0575000	1.000	.753	
	5.1795000	1.000	.740	
	5.3265000	1.000	.727	
	5.3747500	1.000	.714	
	5.3922500	1.000	.701	
	5.3950000	1.000	.688	
	5.3970000	1.000	.675	
	5.4000000	1.000	.662	
	5.4060000	1.000	.649	
	5.4110000	1.000	.636	
	5.4135000	1.000	.623	
	5.4160000	1.000	.610	
	5.4375000	1.000	.597	
	5.4595000	1.000	.584	
	5.4710000	1.000	.571	
	5.4811500	1.000	.558	
	5.4834000	1.000	.545	
	5.4900000	1.000	.532	
	5.4962500	1.000	.519	
	5.5300000	1.000	.506	
	5.6237500	1.000	.494	
	5.7142500	1.000	.481	
	5.7600000	1.000	.468	
	5.7956500	1.000	.455	
	5.8431500	1.000	.442	
	5.9071000	1.000	.429	
	5.9675000	1.000	.416	
	6.1374000	1.000	.403	
	6.3449000	1.000	.390	
	6.4079000	1.000	.377	
	6.4111500	1.000	.364	

Coordinates of the curve	
Test variables	Positive if ≥Ý	Sensitivity	1 －­ specificity	
ki-67#	6.4346500	1.000	.351	
	6.4612500	.929	.351	
	6.4714000	.929	.338	
	6.4805000	.929	.325	
	6.5158500	.929	.312	
	6.5635000	.929	.299	
	6.7315000	.929	.286	
	6.9400000	.929	.273	
	7.1770000	.929	.260	
	7.3805000	.929	.247	
	7.4125000	.929	.234	
	7.4330000	.857	.234	
	7.4560000	.857	.221	
	7.4695500	.857	.208	
	7.4843000	.857	.195	
	7.4974500	.857	.182	
	7.5092000	.857	.169	
	7.5334000	.786	.169	
	7.5519000	.786	.156	
	7.5871500	.786	.143	
	7.6199750	.786	.130	
	7.6673250	.786	.117	
	7.8077000	.786	.104	
	8.1012000	.714	.104	
	8.3500000	.714	.091	
	8.5000000	.643	.091	
	8.7000000	.643	.078	
	9.0500000	.571	.078	
	9.4500000	.500	.078	
	9.8750000	.500	.065	
	10.1750000	.429	.065	
	10.2750000	.357	.065	
	10.3750000	.357	.052	
	10.4500000	.357	.039	
	10.5500000	.357	.026	
	10.6500000	.357	.013	
	10.8000000	.357	.000	
	10.9350000	.286	.000	
	10.9850000	.214	.000	
	11.5000000	.143	.000	
	13.5000000	.071	.000	
	16.0000000	.000	.000	

USE ALL.
COMPUTE filter_$=(PPP=2 or PPP=1).
VARIABLE LABELS filter_$ 'PPP=2 or PPP=1 (FILTER)'.
VALUE LABELS filter_$ 0 'Not Selected' 1 'Selected'.
FORMATS filter_$ (f1.0).
FILTER BY filter_$.
EXECUTE.
ROC Prediction ki67# BY PPP (2)
  /PLOT=CURVE(REFERENCE)
  /PRINT=SE COORDINATES
  /CRITERIA=CUTOFF(INCLUDE) TESTPOS(LARGE) DISTRIBUTION(FREE) CI(95)
  /MISSING=EXCLUDE.


ROC curve


[DATA2] /Users/pengmingzheng/Documents/ggo ct/materials and data.sav


Case processing summary	
PPP	effective N	
正ý的Ä	77	
负º的Ä	26	


Area under the curve (AUC)	
Test variables	Area	Std. error	progressive Sig.	 95% confidence interval 	
				Low limit	Upper limit	
Prediction	.893	.027	.000	.870	.976	
ki-67#	.907	.023	.000	.905	.994	


Coordinates of the curve	
Test variables	Positive if ≥Ý	Sensitivity	1 －­ specificity	
Prediction	.0400000	1.000	1.000	
	1.6630000	.987	1.000	
	2.5530000	.987	.962	
	2.8435000	.974	.962	
	2.8685000	.974	.923	
	2.8970000	.974	.885	
	2.9455000	.974	.846	
	3.0760000	.974	.808	
	3.2135000	.974	.769	
	3.2470000	.974	.731	
	3.2935000	.974	.692	
	3.3392500	.974	.654	
	3.3617500	.974	.615	
	3.3970000	.974	.577	
	3.4155000	.974	.538	
	3.4307500	.974	.500	
	3.4610000	.974	.462	
	3.5047500	.961	.462	
	3.5922500	.961	.423	
	3.6627500	.961	.385	
	3.6932500	.961	.346	
	3.7287500	.948	.346	
	3.7465000	.948	.308	
	3.7997500	.948	.269	
	3.8612500	.935	.269	
	3.8835000	.935	.231	
	3.9195000	.922	.231	
	3.9520000	.909	.231	
	3.9607500	.896	.231	
	3.9685000	.883	.231	
	3.9962500	.870	.231	
	4.0192500	.857	.231	
	4.0242500	.857	.192	
	4.0357500	.857	.154	
	4.0460000	.844	.154	
	4.0512500	.831	.154	
	4.0655000	.818	.154	
	4.0770000	.818	.115	
	4.1007500	.792	.115	
	4.1332500	.779	.115	
	4.1467500	.766	.115	
	4.1510000	.753	.115	
	4.1547500	.740	.115	
	4.1940000	.740	.077	
	4.2805000	.740	.038	
	4.3385000	.727	.038	

Coordinates of the curve	
Test variables	Positive if ≥Ý	Sensitivity	1 －­ specificity	
Prediction	4.3765000	.714	.038	
	4.4130000	.701	.038	
	4.4397500	.688	.038	
	4.4632500	.675	.038	
	4.4817500	.662	.038	
	4.5402500	.649	.038	
	4.5950000	.636	.038	
	4.6215000	.623	.038	
	4.6502500	.623	.000	
	4.6665000	.610	.000	
	4.6995000	.584	.000	
	4.7415000	.571	.000	
	4.7632500	.558	.000	
	4.7932500	.545	.000	
	4.8315000	.532	.000	
	4.8527500	.519	.000	
	4.8557500	.506	.000	
	4.8640000	.494	.000	
	4.8750000	.481	.000	
	4.8862500	.468	.000	
	4.9110000	.455	.000	
	4.9295000	.442	.000	
	4.9377500	.429	.000	
	4.9495000	.416	.000	
	5.0025000	.403	.000	
	5.0575000	.377	.000	
	5.1512500	.364	.000	
	5.2660000	.351	.000	
	5.3265000	.338	.000	
	5.3747500	.325	.000	
	5.4062000	.312	.000	
	5.4394500	.299	.000	
	5.4675000	.286	.000	
	5.4892500	.273	.000	
	5.5032500	.260	.000	
	5.5170000	.247	.000	
	5.5417500	.234	.000	
	5.5587500	.221	.000	
	5.6237500	.208	.000	
	5.7142500	.195	.000	
	5.7737500	.182	.000	
	5.9680000	.169	.000	
	6.1572500	.156	.000	
	6.1997500	.143	.000	
	6.2515000	.130	.000	
	6.5107500	.117	.000	

Coordinates of the cureve	
Test variables	Positive if ≥Ý	Sensitivity	1 －­ specificity	
Prediction	6.8665000	.104	.000	
	7.3857500	.091	.000	
	7.9535000	.078	.000	
	8.1382500	.065	.000	
	8.3535000	.052	.000	
	9.2232500	.039	.000	
	9.9380000	.026	.000	
	10.2270000	.013	.000	
	11.4605000	.000	.000	
ki-67#	.2920000	1.000	1.000	
	1.3130000	1.000	.962	
	1.3390000	1.000	.923	
	1.3545000	1.000	.885	
	1.3760000	1.000	.846	
	1.3946000	1.000	.808	
	1.4051000	1.000	.769	
	1.4185000	1.000	.731	
	1.4318500	1.000	.692	
	1.4753500	1.000	.654	
	1.7103000	1.000	.615	
	2.1148000	.987	.615	
	2.3460000	.987	.577	
	2.4000000	.987	.538	
	2.4355000	.974	.538	
	2.4465000	.974	.500	
	2.6020000	.961	.500	
	2.8080000	.961	.462	
	2.8695000	.961	.423	
	3.0285000	.961	.385	
	3.2410000	.961	.346	
	3.3500000	.961	.308	
	3.4085000	.961	.269	
	3.4155000	.961	.231	
	3.4419500	.961	.192	
	3.4994500	.948	.192	
	3.7135000	.948	.154	
	3.9285000	.935	.154	
	4.0032500	.922	.154	
	4.1947500	.909	.154	
	4.3463500	.909	.115	
	4.3610500	.896	.115	
	4.3797500	.896	.077	
	4.4391000	.883	.077	
	4.5085500	.870	.077	
	4.5370000	.857	.077	
	4.5890000	.844	.077	

Coordinates of the curve	
Test variables	Positive if ≥Ý	Sensitivity	1 －­ specificity	
ki-67#	4.6462500	.831	.077	
	4.7192500	.818	.077	
	4.8262500	.805	.077	
	4.8862500	.792	.077	
	4.9720000	.779	.077	
	5.0575000	.753	.077	
	5.1795000	.740	.077	
	5.3090000	.727	.077	
	5.3415000	.727	.038	
	5.3747500	.714	.038	
	5.3922500	.701	.038	
	5.3950000	.688	.038	
	5.3970000	.675	.038	
	5.4000000	.662	.038	
	5.4060000	.649	.038	
	5.4110000	.636	.038	
	5.4135000	.623	.038	
	5.4160000	.610	.038	
	5.4375000	.597	.038	
	5.4595000	.584	.038	
	5.4628000	.571	.038	
	5.4728000	.571	.000	
	5.4811500	.558	.000	
	5.4834000	.545	.000	
	5.4900000	.532	.000	
	5.4962500	.519	.000	
	5.5300000	.506	.000	
	5.6237500	.494	.000	
	5.7142500	.481	.000	
	5.7600000	.468	.000	
	5.7956500	.455	.000	
	5.8431500	.442	.000	
	5.9071000	.429	.000	
	5.9675000	.416	.000	
	6.1374000	.403	.000	
	6.3449000	.390	.000	
	6.4079000	.377	.000	
	6.4111500	.364	.000	
	6.4409000	.351	.000	
	6.4714000	.338	.000	
	6.4805000	.325	.000	
	6.5158500	.312	.000	
	6.5635000	.299	.000	
	6.7315000	.286	.000	
	6.9400000	.273	.000	
	7.1770000	.260	.000	

Coordinates of the curve	
Test variables	Positive if ≥Ý	Sensitivity	1 －­ specificity	
ki-67#	7.3805000	.247	.000	
	7.4255000	.234	.000	
	7.4560000	.221	.000	
	7.4695500	.208	.000	
	7.4843000	.195	.000	
	7.4974500	.182	.000	
	7.5236000	.169	.000	
	7.5519000	.156	.000	
	7.5871500	.143	.000	
	7.6199750	.130	.000	
	7.6673250	.117	.000	
	8.0065000	.104	.000	
	8.4500000	.091	.000	
	9.1000000	.078	.000	
	9.9750000	.065	.000	
	10.3750000	.052	.000	
	10.4500000	.039	.000	
	10.5500000	.026	.000	
	10.6500000	.013	.000	
	11.7000000	.000	.000	

ROC Diameter TV MAX AVG STD ki67# BY PPP (2)
  /PLOT=CURVE(REFERENCE)
  /PRINT=SE COORDINATES
  /CRITERIA=CUTOFF(INCLUDE) TESTPOS(LARGE) DISTRIBUTION(FREE) CI(95)
  /MISSING=EXCLUDE.


ROC curve


[DATA2] /Users/pengmingzheng/Documents/ggo ct/materials and data.sav


Case processing summary	
PPP	effective N	
Positive	77	
Negative	26	


Area under the curve (AUC)	
Test variables	Area	Std. error	progressive Sig.	 95% confidence interval	
				Lower limit	Upper limit	
Diameter	.801	.048	.000	.708	.895	
TV	.822	.043	.000	.737	.907	
MAX	.890	.037	.000	.817	.963	
AVG	.857	.042	.000	.774	.939	
STD	.901	.030	.000	.843	.959	
ki-67#	.907	.023	.000	.905	.994	


Coordinates of the curve	
Test variables	Positive if ≥Ý	Sensitivity	1 －­ specificity	
Diameter	2.2666700	1.000	1.000	
	3.9500000	1.000	.962	
	4.8166650	1.000	.923	
	5.0833350	1.000	.885	
	5.2333350	.987	.885	
	5.4500000	.987	.808	
	5.7000000	.974	.808	
	6.2333350	.961	.808	
	6.8166700	.935	.808	
	7.0166700	.922	.808	
	7.1000000	.922	.769	
	7.1833300	.922	.731	
	7.2500000	.922	.692	
	7.4333350	.922	.654	
	7.6333350	.922	.577	
	7.6833350	.909	.577	
	7.8166650	.896	.577	
	7.9833300	.883	.577	
	8.1500000	.870	.577	
	8.3666700	.870	.500	
	8.5166700	.870	.462	
	8.6666700	.857	.462	
	8.8666700	.831	.462	
	9.0166700	.831	.423	
	9.3000000	.818	.423	
	9.5666650	.805	.423	
	9.7500000	.805	.385	
	9.9500000	.792	.385	
	10.0166650	.779	.385	
	10.1166650	.779	.346	
	10.2166650	.727	.346	
	10.2500000	.727	.308	
	10.3166700	.727	.269	
	10.4000000	.727	.231	
	10.5500000	.727	.192	
	10.7333350	.714	.192	
	10.8666650	.688	.192	
	11.0500000	.688	.154	
	11.1833350	.675	.154	
	11.2666650	.662	.154	
	11.4333300	.649	.154	
	11.6333300	.636	.115	
	11.8166650	.623	.115	
	12.0833350	.610	.115	
	12.5333350	.597	.115	
	13.0333350	.584	.115	

Coordinates of the curve	
Test variables	Positive if ≥Ý	Sensitivity	1 －­ specificity	
Diameter	13.3166700	.571	.115	
	13.5500000	.558	.115	
	13.9833300	.558	.077	
	14.3666650	.545	.077	
	14.6500000	.532	.077	
	14.8833350	.519	.077	
	15.0000000	.506	.077	
	15.1333300	.481	.077	
	15.5833300	.468	.077	
	16.0166650	.455	.077	
	16.1500000	.442	.077	
	16.2333350	.416	.077	
	16.4666700	.403	.077	
	16.7666700	.390	.077	
	16.9000000	.377	.077	
	16.9666650	.364	.077	
	17.1333350	.351	.077	
	17.3333350	.325	.077	
	17.5666650	.312	.077	
	17.7666650	.299	.077	
	17.9333350	.299	.038	
	18.0833350	.286	.038	
	18.2333350	.273	.038	
	18.3833350	.260	.038	
	18.5500000	.247	.038	
	18.8333350	.234	.038	
	19.0000000	.221	.038	
	19.2666650	.208	.038	
	19.5833350	.208	.000	
	19.7500000	.195	.000	
	20.2166650	.182	.000	
	20.8000000	.169	.000	
	21.2500000	.156	.000	
	21.6166650	.143	.000	
	22.6500000	.130	.000	
	24.0666700	.117	.000	
	26.3333350	.104	.000	
	28.2500000	.091	.000	
	28.4666650	.078	.000	
	29.1333300	.065	.000	
	29.8000000	.052	.000	
	31.5166700	.039	.000	
	33.3333350	.026	.000	
	37.1333350	.013	.000	
	41.7666700	.000	.000	
TV	16.0000000	1.000	1.000	

Coordinates of the curve	
Test variables	Positive if ≥Ý	Sensitivity	1 －­ specificity	
TV	26.5000000	1.000	.962	
	38.0000000	1.000	.923	
	40.5000000	1.000	.885	
	41.5000000	1.000	.846	
	43.5000000	.987	.846	
	54.0000000	.987	.808	
	65.0000000	.974	.808	
	68.5000000	.961	.808	
	73.5000000	.948	.808	
	80.5000000	.935	.808	
	86.5000000	.922	.769	
	89.5000000	.909	.769	
	92.0000000	.909	.731	
	101.5000000	.909	.692	
	110.5000000	.909	.654	
	113.0000000	.909	.615	
	114.5000000	.909	.577	
	121.0000000	.909	.538	
	128.5000000	.896	.538	
	131.5000000	.896	.500	
	134.0000000	.883	.500	
	139.5000000	.883	.462	
	145.5000000	.883	.423	
	147.5000000	.870	.423	
	157.0000000	.831	.423	
	170.5000000	.818	.423	
	178.0000000	.818	.385	
	183.5000000	.805	.385	
	188.5000000	.805	.346	
	194.5000000	.805	.308	
	202.0000000	.805	.269	
	217.0000000	.805	.231	
	233.0000000	.792	.231	
	240.5000000	.779	.231	
	245.5000000	.753	.231	
	255.0000000	.740	.231	
	265.0000000	.727	.231	
	271.0000000	.701	.231	
	278.5000000	.688	.231	
	288.5000000	.675	.231	
	300.0000000	.649	.231	
	307.0000000	.636	.231	
	309.5000000	.636	.192	
	314.0000000	.636	.154	
	321.0000000	.623	.154	
	330.0000000	.623	.115	

Coordinates of the curve	
Test variables	Positive if ≥Ý	Sensitivity	1 －­ specificity	
TV	342.5000000	.610	.115	
	360.5000000	.597	.115	
	372.5000000	.584	.115	
	384.5000000	.584	.077	
	401.0000000	.571	.077	
	407.5000000	.558	.077	
	409.0000000	.545	.077	
	455.0000000	.532	.077	
	513.5000000	.519	.077	
	538.5000000	.506	.077	
	555.5000000	.494	.077	
	581.5000000	.481	.077	
	605.0000000	.468	.077	
	630.0000000	.468	.038	
	669.0000000	.455	.038	
	713.5000000	.442	.038	
	785.0000000	.429	.038	
	847.5000000	.416	.038	
	873.5000000	.403	.038	
	895.5000000	.390	.038	
	924.5000000	.377	.038	
	939.5000000	.364	.038	
	947.0000000	.351	.000	
	963.0000000	.338	.000	
	985.0000000	.325	.000	
	1011.5000000	.312	.000	
	1056.5000000	.299	.000	
	1103.0000000	.286	.000	
	1142.5000000	.273	.000	
	1175.5000000	.260	.000	
	1189.0000000	.247	.000	
	1216.0000000	.234	.000	
	1275.5000000	.221	.000	
	1336.0000000	.208	.000	
	1371.0000000	.195	.000	
	1400.5000000	.182	.000	
	1419.0000000	.169	.000	
	1448.0000000	.156	.000	
	1478.5000000	.143	.000	
	1543.0000000	.130	.000	
	1818.0000000	.117	.000	
	2191.5000000	.104	.000	
	2750.5000000	.091	.000	
	3174.5000000	.078	.000	
	3376.5000000	.065	.000	
	3812.5000000	.052	.000	

Coordinates of the curve	
Test variables	Positive if ≥Ý	Sensitivity	1 －­ specificity	
TV	4342.5000000	.039	.000	
	4838.5000000	.026	.000	
	5116.0000000	.013	.000	
	5169.0000000	.000	.000	
MAX	-601.0000000	1.000	1.000	
	-596.0000000	1.000	.962	
	-585.5000000	.987	.962	
	-569.5000000	.987	.923	
	-512.5000000	.987	.885	
	-464.5000000	.987	.846	
	-451.5000000	.987	.808	
	-435.5000000	.987	.769	
	-404.0000000	.974	.769	
	-373.5000000	.974	.731	
	-369.5000000	.974	.692	
	-364.0000000	.974	.654	
	-341.0000000	.961	.654	
	-320.0000000	.948	.615	
	-310.0000000	.948	.577	
	-301.5000000	.948	.500	
	-298.5000000	.948	.462	
	-290.0000000	.935	.462	
	-280.5000000	.935	.423	
	-273.0000000	.935	.385	
	-250.0000000	.922	.385	
	-212.0000000	.909	.385	
	-191.0000000	.909	.346	
	-187.0000000	.896	.346	
	-181.5000000	.896	.308	
	-175.0000000	.883	.308	
	-171.0000000	.883	.269	
	-167.0000000	.883	.231	
	-157.0000000	.883	.192	
	-149.0000000	.870	.192	
	-139.5000000	.857	.192	
	-126.5000000	.857	.154	
	-120.0000000	.844	.154	
	-116.5000000	.831	.154	
	-112.0000000	.805	.154	
	-108.0000000	.792	.154	
	-98.0000000	.779	.154	
	-78.5000000	.766	.154	
	-67.5000000	.753	.154	
	-64.5000000	.753	.115	
	-61.5000000	.740	.115	
	-60.5000000	.727	.115	

Coordinates of the curve	
Test variables	Positive if ≥Ý	Sensitivity	1 －­ specificity	
MAX	-59.5000000	.714	.115	
	-56.5000000	.701	.115	
	-50.5000000	.688	.115	
	-39.5000000	.675	.115	
	-29.5000000	.675	.077	
	-24.5000000	.675	.038	
	-18.0000000	.662	.038	
	-10.5000000	.649	.038	
	-6.0000000	.636	.038	
	-3.0000000	.623	.038	
	-.5000000	.610	.038	
	3.5000000	.584	.038	
	8.5000000	.571	.038	
	11.0000000	.532	.038	
	15.0000000	.519	.038	
	20.5000000	.506	.038	
	26.0000000	.494	.038	
	31.0000000	.481	.038	
	34.0000000	.468	.038	
	37.0000000	.455	.038	
	40.5000000	.442	.038	
	45.0000000	.429	.038	
	48.5000000	.403	.038	
	49.5000000	.390	.038	
	50.5000000	.377	.038	
	55.0000000	.364	.038	
	61.5000000	.338	.038	
	68.0000000	.325	.038	
	75.0000000	.312	.038	
	83.5000000	.299	.038	
	96.0000000	.286	.038	
	108.0000000	.273	.038	
	135.5000000	.260	.038	
	158.5000000	.247	.038	
	161.5000000	.247	.000	
	166.5000000	.234	.000	
	172.0000000	.221	.000	
	179.5000000	.208	.000	
	184.5000000	.195	.000	
	194.0000000	.182	.000	
	221.0000000	.143	.000	
	246.5000000	.130	.000	
	255.0000000	.104	.000	
	263.0000000	.091	.000	
	279.5000000	.065	.000	
	295.0000000	.052	.000	

Coordinates of the curve	
Test variables	Positive if ≥Ý	Sensitivity	1 －­ specificity	
MAX	326.0000000	.039	.000	
	360.5000000	.026	.000	
	373.5000000	.013	.000	
	378.0000000	.000	.000	
AVG	-758.0000000	1.000	1.000	
	-740.5000000	1.000	.962	
	-713.0000000	1.000	.923	
	-700.5000000	1.000	.885	
	-698.5000000	.987	.885	
	-697.5000000	.974	.885	
	-696.0000000	.974	.846	
	-694.0000000	.974	.769	
	-692.0000000	.961	.731	
	-690.5000000	.961	.692	
	-688.0000000	.961	.654	
	-678.5000000	.961	.615	
	-669.0000000	.961	.577	
	-663.0000000	.961	.538	
	-658.0000000	.948	.538	
	-655.5000000	.935	.538	
	-653.5000000	.922	.538	
	-652.5000000	.909	.538	
	-651.5000000	.909	.500	
	-650.0000000	.896	.500	
	-647.5000000	.883	.500	
	-645.5000000	.883	.462	
	-644.5000000	.844	.462	
	-643.0000000	.844	.423	
	-641.0000000	.844	.385	
	-639.0000000	.844	.346	
	-636.5000000	.844	.308	
	-634.0000000	.844	.269	
	-632.0000000	.831	.269	
	-630.5000000	.818	.269	
	-628.5000000	.792	.231	
	-626.5000000	.792	.192	
	-623.0000000	.779	.192	
	-618.5000000	.779	.154	
	-615.5000000	.779	.115	
	-613.0000000	.753	.115	
	-611.5000000	.740	.115	
	-610.5000000	.727	.115	
	-609.5000000	.714	.115	
	-608.5000000	.714	.077	
	-606.5000000	.701	.077	
	-603.0000000	.688	.077	

Coordinates of the curve	
Test variables	Positive if ≥Ý	Sensitivity	1 －­ specificity	
AVG	-600.0000000	.675	.077	
	-597.0000000	.662	.077	
	-594.5000000	.649	.077	
	-593.5000000	.623	.077	
	-591.5000000	.597	.077	
	-589.5000000	.571	.077	
	-588.0000000	.558	.077	
	-585.0000000	.545	.077	
	-581.5000000	.545	.038	
	-576.0000000	.519	.038	
	-571.0000000	.506	.038	
	-569.5000000	.481	.038	
	-568.0000000	.468	.038	
	-566.5000000	.455	.038	
	-565.5000000	.442	.038	
	-564.5000000	.429	.038	
	-562.5000000	.416	.038	
	-560.0000000	.403	.038	
	-558.5000000	.390	.038	
	-556.0000000	.377	.038	
	-552.5000000	.364	.038	
	-550.5000000	.338	.038	
	-548.5000000	.325	.038	
	-544.0000000	.312	.038	
	-540.0000000	.299	.038	
	-538.0000000	.286	.038	
	-536.0000000	.273	.038	
	-534.5000000	.260	.038	
	-533.5000000	.247	.038	
	-532.5000000	.221	.038	
	-530.0000000	.208	.038	
	-527.0000000	.195	.038	
	-525.5000000	.169	.038	
	-523.0000000	.156	.038	
	-520.5000000	.156	.000	
	-518.5000000	.143	.000	
	-512.5000000	.117	.000	
	-507.5000000	.091	.000	
	-505.5000000	.078	.000	
	-499.5000000	.065	.000	
	-488.5000000	.052	.000	
	-476.0000000	.039	.000	
	-459.5000000	.026	.000	
	-445.5000000	.013	.000	
	-441.0000000	.000	.000	
STD	22.4000000	1.000	1.000	

Coordinates of the curve	
Test variables	Positive if ≥Ý	Sensitivity	1 －­ specificity	
STD	29.8000000	1.000	.962	
	38.4000000	.987	.962	
	47.1000000	.987	.923	
	54.6000000	.987	.885	
	60.8000000	.987	.846	
	67.2000000	.987	.808	
	69.1500000	.987	.769	
	70.2000000	.987	.731	
	72.0500000	.987	.692	
	74.7000000	.987	.654	
	76.8000000	.987	.615	
	78.3000000	.987	.577	
	80.8500000	.987	.538	
	83.8000000	.987	.500	
	86.6500000	.974	.500	
	89.3500000	.961	.500	
	90.1500000	.948	.500	
	91.8500000	.948	.462	
	93.6000000	.935	.462	
	93.9500000	.922	.462	
	94.6500000	.922	.423	
	95.7500000	.909	.423	
	96.8000000	.896	.423	
	97.4000000	.896	.385	
	97.6500000	.870	.385	
	100.9500000	.857	.385	
	104.8000000	.844	.385	
	107.2000000	.831	.385	
	109.2000000	.831	.308	
	109.8500000	.818	.308	
	110.5000000	.818	.269	
	110.7500000	.805	.269	
	111.0500000	.805	.231	
	111.3500000	.792	.231	
	113.6000000	.792	.192	
	116.0500000	.779	.192	
	117.2500000	.779	.154	
	118.3000000	.766	.154	
	122.6500000	.766	.115	
	127.1500000	.753	.115	
	127.7000000	.740	.115	
	128.3500000	.740	.077	
	128.8500000	.727	.077	
	131.8000000	.727	.038	
	134.8500000	.714	.038	
	135.5000000	.714	.000	

Coordinates of the curve	
Test variables	Positive if ≥Ý	Sensitivity	1 －­ specificity	
STD	136.1500000	.701	.000	
	136.8000000	.688	.000	
	139.4500000	.675	.000	
	142.9000000	.662	.000	
	144.5500000	.649	.000	
	145.1000000	.636	.000	
	145.3500000	.623	.000	
	145.9000000	.597	.000	
	147.1500000	.584	.000	
	148.2500000	.571	.000	
	148.7000000	.558	.000	
	149.1000000	.545	.000	
	151.3000000	.532	.000	
	154.7500000	.506	.000	
	157.5000000	.494	.000	
	159.5500000	.481	.000	
	160.8000000	.468	.000	
	162.2000000	.455	.000	
	163.3500000	.442	.000	
	164.4000000	.416	.000	
	166.1000000	.403	.000	
	168.0500000	.390	.000	
	169.9500000	.364	.000	
	170.8500000	.338	.000	
	171.8000000	.325	.000	
	175.2000000	.312	.000	
	180.5000000	.299	.000	
	183.7500000	.286	.000	
	186.6000000	.273	.000	
	190.1500000	.260	.000	
	191.7500000	.247	.000	
	192.6000000	.234	.000	
	193.8500000	.221	.000	
	195.4500000	.208	.000	
	197.4500000	.195	.000	
	199.6000000	.182	.000	
	202.5000000	.169	.000	
	205.3500000	.143	.000	
	206.2500000	.130	.000	
	206.7000000	.117	.000	
	209.1000000	.091	.000	
	211.7000000	.078	.000	
	212.4500000	.065	.000	
	213.7000000	.052	.000	
	216.5500000	.039	.000	
	222.5500000	.026	.000	

Coordinate of the curve	
Test variables	Positive if ≥Ý	Sensitivity	1 －­ specificity	
STD	234.4000000	.013	.000	
	243.0000000	.000	.000	
ki-67#	.2920000	1.000	1.000	
	1.3130000	1.000	.962	
	1.3390000	1.000	.923	
	1.3545000	1.000	.885	
	1.3760000	1.000	.846	
	1.3946000	1.000	.808	
	1.4051000	1.000	.769	
	1.4185000	1.000	.731	
	1.4318500	1.000	.692	
	1.4753500	1.000	.654	
	1.7103000	1.000	.615	
	2.1148000	.987	.615	
	2.3460000	.987	.577	
	2.4000000	.987	.538	
	2.4355000	.974	.538	
	2.4465000	.974	.500	
	2.6020000	.961	.500	
	2.8080000	.961	.462	
	2.8695000	.961	.423	
	3.0285000	.961	.385	
	3.2410000	.961	.346	
	3.3500000	.961	.308	
	3.4085000	.961	.269	
	3.4155000	.961	.231	
	3.4419500	.961	.192	
	3.4994500	.948	.192	
	3.7135000	.948	.154	
	3.9285000	.935	.154	
	4.0032500	.922	.154	
	4.1947500	.909	.154	
	4.3463500	.909	.115	
	4.3610500	.896	.115	
	4.3797500	.896	.077	
	4.4391000	.883	.077	
	4.5085500	.870	.077	
	4.5370000	.857	.077	
	4.5890000	.844	.077	
	4.6462500	.831	.077	
	4.7192500	.818	.077	
	4.8262500	.805	.077	
	4.8862500	.792	.077	
	4.9720000	.779	.077	
	5.0575000	.753	.077	
	5.1795000	.740	.077	

Coordinate of the curve	
Test variables	Positive if ≥Ý	Sensitivity	1 －­ specificity	
ki-67#	5.3090000	.727	.077	
	5.3415000	.727	.038	
	5.3747500	.714	.038	
	5.3922500	.701	.038	
	5.3950000	.688	.038	
	5.3970000	.675	.038	
	5.4000000	.662	.038	
	5.4060000	.649	.038	
	5.4110000	.636	.038	
	5.4135000	.623	.038	
	5.4160000	.610	.038	
	5.4375000	.597	.038	
	5.4595000	.584	.038	
	5.4628000	.571	.038	
	5.4728000	.571	.000	
	5.4811500	.558	.000	
	5.4834000	.545	.000	
	5.4900000	.532	.000	
	5.4962500	.519	.000	
	5.5300000	.506	.000	
	5.6237500	.494	.000	
	5.7142500	.481	.000	
	5.7600000	.468	.000	
	5.7956500	.455	.000	
	5.8431500	.442	.000	
	5.9071000	.429	.000	
	5.9675000	.416	.000	
	6.1374000	.403	.000	
	6.3449000	.390	.000	
	6.4079000	.377	.000	
	6.4111500	.364	.000	
	6.4409000	.351	.000	
	6.4714000	.338	.000	
	6.4805000	.325	.000	
	6.5158500	.312	.000	
	6.5635000	.299	.000	
	6.7315000	.286	.000	
	6.9400000	.273	.000	
	7.1770000	.260	.000	
	7.3805000	.247	.000	
	7.4255000	.234	.000	
	7.4560000	.221	.000	
	7.4695500	.208	.000	
	7.4843000	.195	.000	
	7.4974500	.182	.000	
	7.5236000	.169	.000	

Coordinate of the curve	
Test variables	Positive if ≥Ý	Sensitivity	1 －­ specificity	
ki-67#	7.5519000	.156	.000	
	7.5871500	.143	.000	
	7.6199750	.130	.000	
	7.6673250	.117	.000	
	8.0065000	.104	.000	
	8.4500000	.091	.000	
	9.1000000	.078	.000	
	9.9750000	.065	.000	
	10.3750000	.052	.000	
	10.4500000	.039	.000	
	10.5500000	.026	.000	
	10.6500000	.013	.000	
	11.7000000	.000	.000	

USE ALL.
COMPUTE filter_$=(PPP=2 or PPP=3).
VARIABLE LABELS filter_$ 'PPP=2 or PPP=3 (FILTER)'.
VALUE LABELS filter_$ 0 'Not Selected' 1 'Selected'.
FORMATS filter_$ (f1.0).
FILTER BY filter_$.
EXECUTE.
ROC Diameter TV MAX AVG STD ki67# BY PPP (3)
  /PLOT=CURVE(REFERENCE)
  /PRINT=SE COORDINATES
  /CRITERIA=CUTOFF(INCLUDE) TESTPOS(LARGE) DISTRIBUTION(FREE) CI(95)
  /MISSING=EXCLUDE.


ROC curve


[DATA2] /Users/pengmingzheng/Documents/ggo ct/materials and data.sav


Case processing summary	
PPP	effective N	
Positive	14	
Negative	77	


Area under the curve	
Test variables	Area	Std. error	progressive Sig.	 95% confidence interval	
				Lower limit	Upper limit	
Diameter	.766	.069	.002	.630	.902	
TV	.749	.072	.003	.607	.891	
MAX	.702	.091	.017	.524	.880	
AVG	.690	.090	.024	.514	.866	
STD	.650	.088	.075	.477	.823	
ki-67#	.912	.034	.000	.846	.978	


Coordinate of the curve	
Test variables	Positive if ≥Ý	Sensitivity	1 －­ specificity	
Diameter	4.1666700	1.000	1.000	
	5.3833350	1.000	.987	
	5.7000000	1.000	.974	
	6.2333350	1.000	.961	
	6.8166700	1.000	.935	
	7.3166700	1.000	.922	
	7.6833350	1.000	.909	
	7.8166650	1.000	.896	
	7.9833300	1.000	.883	
	8.3000000	1.000	.870	
	8.6666700	1.000	.857	
	8.8833350	1.000	.831	
	9.0333350	.929	.831	
	9.3000000	.929	.818	
	9.7166650	.929	.805	
	9.9500000	.929	.792	
	10.1000000	.929	.779	
	10.4333350	.929	.727	
	10.7333350	.929	.714	
	10.9833350	.929	.688	
	11.1833350	.929	.675	
	11.2666650	.929	.662	
	11.4333300	.929	.649	
	11.6333300	.929	.636	
	11.8166650	.929	.623	
	12.0833350	.929	.610	
	12.5333350	.929	.597	
	12.8500000	.929	.584	
	13.0833350	.857	.584	
	13.3166700	.857	.571	
	13.8000000	.857	.558	
	14.3666650	.857	.545	
	14.6500000	.857	.532	
	14.8833350	.857	.519	
	15.0000000	.857	.506	
	15.1333300	.857	.481	
	15.5500000	.857	.468	
	15.9000000	.786	.468	
	16.0166650	.786	.455	
	16.1500000	.786	.442	
	16.2333350	.786	.416	
	16.4666700	.786	.403	
	16.7666700	.786	.390	
	16.9000000	.786	.377	
	16.9666650	.786	.364	
	17.0166650	.786	.351	

Coordinate of the curve	
Test vairbales	Positive if ≥Ý	Sensitivity	1 －­ specificity	
Diameter	17.1500000	.714	.351	
	17.3333350	.714	.325	
	17.4833350	.714	.312	
	17.6500000	.643	.312	
	17.9000000	.643	.299	
	18.0833350	.643	.286	
	18.2333350	.643	.273	
	18.3833350	.643	.260	
	18.5500000	.643	.247	
	18.8333350	.643	.234	
	19.0000000	.643	.221	
	19.3500000	.643	.208	
	19.7500000	.643	.195	
	20.2166650	.643	.182	
	20.8000000	.643	.169	
	21.2500000	.643	.156	
	21.6166650	.643	.143	
	21.8000000	.643	.130	
	22.1666700	.571	.130	
	23.0166700	.500	.130	
	24.0000000	.500	.117	
	24.5000000	.429	.117	
	25.1166700	.429	.104	
	26.5833350	.357	.104	
	27.5500000	.286	.104	
	27.8500000	.214	.104	
	28.2500000	.214	.091	
	28.4666650	.214	.078	
	29.1333300	.214	.065	
	29.8000000	.214	.052	
	31.5166700	.214	.039	
	33.3333350	.143	.026	
	34.6166650	.143	.013	
	38.2500000	.071	.013	
	41.9833350	.071	.000	
	44.2000000	.000	.000	
TV	41.0000000	1.000	1.000	
	52.5000000	1.000	.987	
	65.0000000	1.000	.974	
	68.5000000	1.000	.961	
	73.5000000	1.000	.948	
	80.5000000	1.000	.935	
	86.5000000	1.000	.922	
	108.0000000	1.000	.909	
	130.0000000	1.000	.896	
	140.0000000	1.000	.883	

Coordinate of the curve	
Test variables	Positive if ≥Ý	Sensitivity	1 －­ specificity	
TV	147.5000000	1.000	.870	
	153.5000000	1.000	.831	
	162.5000000	.929	.831	
	173.5000000	.929	.818	
	204.5000000	.929	.805	
	233.0000000	.929	.792	
	240.5000000	.929	.779	
	245.5000000	.929	.753	
	255.0000000	.929	.740	
	265.0000000	.929	.727	
	271.0000000	.929	.701	
	278.5000000	.929	.688	
	288.5000000	.929	.675	
	300.0000000	.929	.649	
	311.5000000	.929	.636	
	326.0000000	.929	.623	
	342.5000000	.929	.610	
	350.5000000	.929	.597	
	361.0000000	.857	.597	
	383.0000000	.857	.584	
	401.0000000	.857	.571	
	407.5000000	.857	.558	
	409.0000000	.857	.545	
	455.0000000	.857	.532	
	513.5000000	.857	.519	
	538.5000000	.857	.506	
	555.5000000	.857	.494	
	578.0000000	.857	.481	
	598.5000000	.786	.481	
	627.0000000	.786	.468	
	669.0000000	.786	.455	
	713.5000000	.786	.442	
	779.0000000	.786	.429	
	823.0000000	.714	.429	
	837.0000000	.714	.416	
	855.5000000	.643	.416	
	873.5000000	.643	.403	
	895.5000000	.643	.390	
	924.5000000	.643	.377	
	939.5000000	.643	.364	
	947.0000000	.643	.351	
	963.0000000	.643	.338	
	985.0000000	.643	.325	
	1011.5000000	.643	.312	
	1035.5000000	.643	.299	
	1067.0000000	.571	.299	

Coordinate of the curve	
Test variables	Positive if ≥Ý	Sensitivity	1 －­ specificity	
TV	1103.0000000	.571	.286	
	1142.5000000	.571	.273	
	1175.5000000	.571	.260	
	1189.0000000	.571	.247	
	1216.0000000	.571	.234	
	1275.5000000	.571	.221	
	1336.0000000	.571	.208	
	1371.0000000	.571	.195	
	1400.5000000	.571	.182	
	1419.0000000	.571	.169	
	1448.0000000	.571	.156	
	1478.5000000	.571	.143	
	1543.0000000	.571	.130	
	1708.5000000	.571	.117	
	1921.5000000	.500	.117	
	2067.0000000	.500	.104	
	2227.5000000	.429	.104	
	2426.5000000	.429	.091	
	2825.0000000	.357	.091	
	3174.5000000	.357	.078	
	3242.5000000	.357	.065	
	3419.0000000	.286	.065	
	3618.5000000	.286	.052	
	3878.0000000	.214	.052	
	4217.5000000	.214	.039	
	4488.0000000	.143	.039	
	4838.5000000	.143	.026	
	5116.0000000	.143	.013	
	6521.5000000	.143	.000	
	7927.0000000	.071	.000	
	7980.0000000	.000	.000	
MAX	-593.0000000	1.000	1.000	
	-523.0000000	1.000	.987	
	-443.0000000	.929	.987	
	-396.0000000	.929	.974	
	-341.0000000	.929	.961	
	-309.0000000	.929	.948	
	-282.5000000	.929	.935	
	-250.0000000	.929	.922	
	-227.5000000	.929	.909	
	-206.5000000	.857	.909	
	-183.5000000	.857	.896	
	-164.0000000	.857	.883	
	-149.0000000	.857	.870	
	-135.0000000	.857	.857	
	-120.0000000	.857	.844	

Coordinate of the curve	
Test variables	Positive if ≥Ý	Sensitivity	1 －­ specificity	
MAX	-116.5000000	.857	.831	
	-112.0000000	.857	.805	
	-108.0000000	.857	.792	
	-98.0000000	.857	.779	
	-78.5000000	.857	.766	
	-65.0000000	.857	.753	
	-61.5000000	.857	.740	
	-60.5000000	.857	.727	
	-59.5000000	.857	.714	
	-56.5000000	.857	.701	
	-50.5000000	.857	.688	
	-34.5000000	.857	.675	
	-18.0000000	.786	.662	
	-10.5000000	.786	.649	
	-6.0000000	.786	.636	
	-3.0000000	.786	.623	
	-.5000000	.786	.610	
	3.5000000	.786	.584	
	8.5000000	.786	.571	
	11.0000000	.786	.532	
	15.0000000	.786	.519	
	20.5000000	.786	.506	
	24.0000000	.786	.494	
	27.0000000	.714	.494	
	31.0000000	.714	.481	
	34.0000000	.714	.468	
	37.0000000	.714	.455	
	40.5000000	.714	.442	
	45.0000000	.714	.429	
	48.5000000	.714	.403	
	49.5000000	.714	.390	
	50.5000000	.714	.377	
	55.0000000	.714	.364	
	61.5000000	.643	.338	
	68.0000000	.643	.325	
	75.0000000	.643	.312	
	83.5000000	.643	.299	
	92.0000000	.643	.286	
	99.0000000	.571	.286	
	108.0000000	.571	.273	
	135.5000000	.571	.260	
	161.0000000	.571	.247	
	166.5000000	.571	.234	
	172.0000000	.571	.221	
	179.5000000	.571	.208	
	184.5000000	.571	.195	

Coordinate of the curve	
Test vairbales	Positive if ≥Ý	Sensitivity	1 －­ specificity	
MAX	189.0000000	.571	.182	
	198.0000000	.500	.182	
	211.0000000	.500	.143	
	229.0000000	.429	.143	
	246.5000000	.429	.130	
	255.0000000	.429	.104	
	263.0000000	.429	.091	
	275.5000000	.429	.065	
	285.0000000	.357	.065	
	291.0000000	.357	.052	
	297.0000000	.286	.052	
	309.5000000	.286	.039	
	334.5000000	.214	.039	
	360.5000000	.214	.026	
	373.5000000	.214	.013	
	486.5000000	.214	.000	
	641.0000000	.143	.000	
	923.0000000	.071	.000	
	1161.0000000	.000	.000	
AVG	-731.0000000	1.000	1.000	
	-714.5000000	.929	1.000	
	-698.5000000	.929	.987	
	-695.5000000	.929	.974	
	-676.0000000	.929	.961	
	-658.0000000	.929	.948	
	-655.5000000	.929	.935	
	-653.5000000	.929	.922	
	-652.0000000	.929	.909	
	-650.0000000	.929	.896	
	-647.0000000	.929	.883	
	-644.5000000	.929	.844	
	-638.5000000	.857	.844	
	-632.0000000	.857	.831	
	-630.5000000	.857	.818	
	-628.0000000	.857	.792	
	-620.0000000	.857	.779	
	-613.0000000	.857	.753	
	-611.5000000	.857	.740	
	-610.5000000	.857	.727	
	-609.0000000	.857	.714	
	-606.5000000	.857	.701	
	-603.0000000	.857	.688	
	-600.0000000	.857	.675	
	-597.0000000	.857	.662	
	-594.5000000	.857	.649	
	-593.5000000	.857	.623	

Coordinate of the curve	
Test variables	Positive if ≥Ý	Sensitivity	1 －­ specificity	
AVG	-592.0000000	.857	.597	
	-590.5000000	.786	.597	
	-589.5000000	.786	.571	
	-588.0000000	.786	.558	
	-583.5000000	.786	.545	
	-579.5000000	.786	.519	
	-575.5000000	.714	.519	
	-571.0000000	.714	.506	
	-569.5000000	.714	.481	
	-568.0000000	.714	.468	
	-566.5000000	.714	.455	
	-565.5000000	.714	.442	
	-564.5000000	.643	.429	
	-562.5000000	.643	.416	
	-560.5000000	.643	.403	
	-559.5000000	.571	.403	
	-558.5000000	.571	.390	
	-556.0000000	.571	.377	
	-552.5000000	.571	.364	
	-550.5000000	.571	.338	
	-548.5000000	.571	.325	
	-544.0000000	.571	.312	
	-540.0000000	.571	.299	
	-538.5000000	.571	.286	
	-537.5000000	.500	.286	
	-536.0000000	.500	.273	
	-534.5000000	.500	.260	
	-533.5000000	.500	.247	
	-532.5000000	.500	.221	
	-530.0000000	.500	.208	
	-527.0000000	.429	.195	
	-525.5000000	.429	.169	
	-522.5000000	.429	.156	
	-518.5000000	.429	.143	
	-512.5000000	.429	.117	
	-507.5000000	.429	.091	
	-505.5000000	.429	.078	
	-499.5000000	.429	.065	
	-488.5000000	.429	.052	
	-476.0000000	.429	.039	
	-464.0000000	.429	.026	
	-456.5000000	.357	.026	
	-452.0000000	.286	.026	
	-445.5000000	.286	.013	
	-438.0000000	.286	.000	
	-424.0000000	.214	.000	

Coordinate of the curve	
Test variables	Positive if ≥Ý	Sensitivity	1 －­ specificity	
AVG	-391.0000000	.143	.000	
	-367.0000000	.071	.000	
	-365.0000000	.000	.000	
STD	35.2000000	1.000	1.000	
	46.8500000	1.000	.987	
	71.1000000	.929	.987	
	86.6500000	.929	.974	
	89.3500000	.929	.961	
	91.8000000	.929	.948	
	93.6000000	.929	.935	
	94.4000000	.929	.922	
	95.7500000	.929	.909	
	97.0000000	.929	.896	
	97.6500000	.929	.870	
	100.9500000	.929	.857	
	104.8000000	.929	.844	
	107.4000000	.929	.831	
	110.0500000	.929	.818	
	111.0000000	.929	.805	
	112.2000000	.929	.792	
	114.4500000	.857	.792	
	116.1500000	.857	.779	
	117.3500000	.786	.779	
	122.5500000	.786	.766	
	127.1500000	.786	.753	
	128.0500000	.786	.740	
	131.6500000	.786	.727	
	135.2500000	.786	.714	
	136.1500000	.786	.701	
	136.8000000	.786	.688	
	139.4500000	.786	.675	
	142.9000000	.786	.662	
	144.5500000	.786	.649	
	145.1000000	.786	.636	
	145.3500000	.786	.623	
	145.9000000	.786	.597	
	147.1500000	.786	.584	
	148.2500000	.786	.571	
	148.7000000	.786	.558	
	149.1000000	.786	.545	
	151.1000000	.786	.532	
	153.1000000	.714	.532	
	154.7500000	.714	.506	
	157.5000000	.714	.494	
	158.8500000	.714	.481	
	159.6000000	.643	.481	

Coordinate of the curve	
Test variables	Positive if ≥Ý	Sensitivity	1 －­ specificity	
STD	160.8000000	.643	.468	
	162.2000000	.643	.455	
	163.3500000	.643	.442	
	164.4000000	.643	.416	
	166.1000000	.643	.403	
	168.0500000	.643	.390	
	169.4000000	.643	.364	
	170.2500000	.571	.364	
	170.8500000	.571	.338	
	171.8000000	.571	.325	
	175.2000000	.571	.312	
	179.0000000	.571	.299	
	181.8000000	.500	.299	
	183.7500000	.500	.286	
	186.6000000	.500	.273	
	189.7000000	.500	.260	
	190.5000000	.429	.260	
	190.9500000	.357	.260	
	191.7500000	.357	.247	
	192.6000000	.357	.234	
	193.8500000	.357	.221	
	195.4500000	.357	.208	
	197.4500000	.357	.195	
	199.6000000	.357	.182	
	202.5000000	.357	.169	
	205.3500000	.357	.143	
	206.2500000	.357	.130	
	206.7000000	.357	.117	
	209.1000000	.357	.091	
	211.2000000	.357	.078	
	211.8000000	.286	.078	
	212.4500000	.286	.065	
	213.7000000	.286	.052	
	216.1000000	.286	.039	
	217.8500000	.214	.039	
	221.3000000	.214	.026	
	225.5500000	.143	.026	
	234.4000000	.143	.013	
	252.9500000	.143	.000	
	282.7000000	.071	.000	
	302.5000000	.000	.000	
ki-67#	.9046000	1.000	1.000	
	2.1688000	1.000	.987	
	2.4440000	1.000	.974	
	2.9609500	1.000	.961	
	3.6809500	1.000	.948	

Coordinate of the curve	
Test variables 	Positive if ≥Ý	Sensitivity	1 －­ specificity	
ki-67#	3.9285000	1.000	.935	
	4.0032500	1.000	.922	
	4.1961000	1.000	.909	
	4.3664000	1.000	.896	
	4.4391000	1.000	.883	
	4.5085500	1.000	.870	
	4.5370000	1.000	.857	
	4.5890000	1.000	.844	
	4.6462500	1.000	.831	
	4.7192500	1.000	.818	
	4.8262500	1.000	.805	
	4.8862500	1.000	.792	
	4.9720000	1.000	.779	
	5.0575000	1.000	.753	
	5.1795000	1.000	.740	
	5.3265000	1.000	.727	
	5.3747500	1.000	.714	
	5.3922500	1.000	.701	
	5.3950000	1.000	.688	
	5.3970000	1.000	.675	
	5.4000000	1.000	.662	
	5.4060000	1.000	.649	
	5.4110000	1.000	.636	
	5.4135000	1.000	.623	
	5.4160000	1.000	.610	
	5.4375000	1.000	.597	
	5.4595000	1.000	.584	
	5.4710000	1.000	.571	
	5.4811500	1.000	.558	
	5.4834000	1.000	.545	
	5.4900000	1.000	.532	
	5.4962500	1.000	.519	
	5.5300000	1.000	.506	
	5.6237500	1.000	.494	
	5.7142500	1.000	.481	
	5.7600000	1.000	.468	
	5.7956500	1.000	.455	
	5.8431500	1.000	.442	
	5.9071000	1.000	.429	
	5.9675000	1.000	.416	
	6.1374000	1.000	.403	
	6.3449000	1.000	.390	
	6.4079000	1.000	.377	
	6.4111500	1.000	.364	
	6.4346500	1.000	.351	
	6.4612500	.929	.351	

Coordinate of the curve	
Test variables	Positive if ≥Ý	Sensitivity	1 －­ specificity	
ki-67#	6.4714000	.929	.338	
	6.4805000	.929	.325	
	6.5158500	.929	.312	
	6.5635000	.929	.299	
	6.7315000	.929	.286	
	6.9400000	.929	.273	
	7.1770000	.929	.260	
	7.3805000	.929	.247	
	7.4125000	.929	.234	
	7.4330000	.857	.234	
	7.4560000	.857	.221	
	7.4695500	.857	.208	
	7.4843000	.857	.195	
	7.4974500	.857	.182	
	7.5092000	.857	.169	
	7.5334000	.786	.169	
	7.5519000	.786	.156	
	7.5871500	.786	.143	
	7.6199750	.786	.130	
	7.6673250	.786	.117	
	7.8077000	.786	.104	
	8.1012000	.714	.104	
	8.3500000	.714	.091	
	8.5000000	.643	.091	
	8.7000000	.643	.078	
	9.0500000	.571	.078	
	9.4500000	.500	.078	
	9.8750000	.500	.065	
	10.1750000	.429	.065	
	10.2750000	.357	.065	
	10.3750000	.357	.052	
	10.4500000	.357	.039	
	10.5500000	.357	.026	
	10.6500000	.357	.013	
	10.8000000	.357	.000	
	10.9350000	.286	.000	
	10.9850000	.214	.000	
	11.5000000	.143	.000	
	13.5000000	.071	.000	
	16.0000000	.000	.000	

CORRELATIONS
  /VARIABLES=MAX AVG STD Ki67# CVprediction Diameter TV
  /PRINT=TWOTAIL NOSIG
  /MISSING=PAIRWISE.


Correlation

 [DATA4] /Users/pengmingzheng/Documents/ggo ct/materials and data.sav


Correlation	
	MAX	AVG	STD	Ki-67#	CV-prediction	Diameter	TV	
MAX	Pearson correlation	1	.757	.757	.548	.778	.725	.636	
	Sig.（¨two-tailed）©		.000	.000	.000	.000	.000	.000	
	N	117	117	117	113	117	117	117	
AVG	Pearson correlation	.757	1	.838	.540	.763	.622	.553	
	Sig.（¨two-tailed）©	.000		.000	.000	.000	.000	.000	
	N	117	117	117	113	117	117	117	
STD	Pearson correlation	.757	.838	1	.566	.847	.677	.558	
	Sig.（¨two-tailed）©	.000	.000		.000	.000	.000	.000	
	N	117	117	117	113	117	117	117	
Ki-67#	Pearson correlation	.548	.540	.566	1	.614	.557	.530	
	Sig.（¨two-tailed）©	.000	.000	.000		.000	.000	.000	
	N	113	113	113	113	113	113	113	
CV-prediction	Pearson correlation	.778	.763	.847	.614	1	.901	.911	
	Sig.（¨two-tailed）©	.000	.000	.000	.000		.000	.000	
	N	117	117	117	113	117	117	117	
Diameter	Pearson correlation	.725	.622	.677	.557	.901	1	.886	
	Sig.（¨two-tailed）©	.000	.000	.000	.000	.000		.000	
	N	117	117	117	113	117	117	117	
TV	Pearson correlation	.636	.553	.558	.530	.911	.886	1	
	Sig.（¨two-tailed）©	.000	.000	.000	.000	.000	.000		
	N	117	117	117	113	117	117	117	

NONPAR CORR
  /VARIABLES=MAX AVG STD Ki67# CVprediction Diameter TV
  /PRINT=SPEARMAN TWOTAIL NOSIG
  /MISSING=PAIRWISE.


Non-parametric correlation coefficient


[DATA4] /Users/pengmingzheng/Documents/ggo ct/materials and data.sav


Correlation coefficient	
	MAX	AVG	STD	Ki-67#	CV-prediction	Diameter	
Spearman's rho	MAX	Coefficient	1.000	.752	.801	.553	.876	.788	
		Sig.	.	.000	.000	.000	.000	.000	
		N	117	117	117	113	117	117	
	AVG	Coefficient	.752	1.000	.829	.511	.804	.589	
		Sig.	.000	.	.000	.000	.000	.000	
		N	117	117	117	113	117	117	
	STD	Coefficient	.801	.829	1.000	.590	.923	.643	
		Sig.	.000	.000	.	.000	.000	.000	
		N	117	117	117	113	117	117	
	Ki-67#	Coefficient	.553	.511	.590	1.000	.617	.566	
		Sig.	.000	.000	.000	.	.000	.000	
		N	113	113	113	113	113	113	
	CV-prediction	Coefficient	.876	.804	.923	.617	1.000	.836	
		Sig.	.000	.000	.000	.000	.	.000	
		N	117	117	117	113	117	117	
	Diameter	Coefficient	.788	.589	.643	.566	.836	1.000	
		Sig.	.000	.000	.000	.000	.000	.	
		N	117	117	117	113	117	117	
	TV	Coefficient	.748	.587	.594	.560	.818	.960	
		Sig.	.000	.000	.000	.000	.000	.000	
		N	117	117	117	113	117	117	

Correlation coefficient	
	TV	
Spearman's rho	MAX	Coefficient	.748	
		Sig.	.000	
		N	117	
	AVG	Coefficient	.587	
		Sig.	.000	
		N	117	
	STD	Coefficient	.594	
		Sig.	.000	
		N	117	
	Ki-67#	Coefficient	.560	
		Sig.	.000	
		N	113	
	CV-prediction	Coefficient	.818	
		Sig.	.000	
		N	117	
	Diameter	Coefficient	.960	
		Sig.	.000	
		N	117	
	TV	Coefficient	1.000	
		Sig.	.	
		N	117	

CORRELATIONS
  /VARIABLES=MAX AVG STD Ki67# CVprediction Diameter TV
  /PRINT=ONETAIL NOSIG
  /MISSING=PAIRWISE.


correlation


 [DATA4] /Users/pengmingzheng/Documents/ggo ct/materials and data.sav


Correlation	
	MAX	AVG	STD	Ki-67#	CV-prediction	Diameter	TV	
MAX	Pearson correlation	1	.757	.757	.548	.778	.725	.636	
	Sig.		.000	.000	.000	.000	.000	.000	
	N	117	117	117	113	117	117	117	
AVG	Pearson correlation	.757	1	.838	.540	.763	.622	.553	
	Sig. 	.000		.000	.000	.000	.000	.000	
	N	117	117	117	113	117	117	117	
STD	Pearson correlation	.757	.838	1	.566	.847	.677	.558	
	Sig. 	.000	.000		.000	.000	.000	.000	
	N	117	117	117	113	117	117	117	
Ki-67#	Pearson correlation	.548	.540	.566	1	.614	.557	.530	
	Sig.	.000	.000	.000		.000	.000	.000	
	N	113	113	113	113	113	113	113	
CV-prediction	Pearson correlation	.778	.763	.847	.614	1	.901	.911	
	Sig.	.000	.000	.000	.000		.000	.000	
	N	117	117	117	113	117	117	117	
Diameter	Pearson correlation	.725	.622	.677	.557	.901	1	.886	
	Sig.	.000	.000	.000	.000	.000		.000	
	N	117	117	117	113	117	117	117	
TV	Pearson correlation	.636	.553	.558	.530	.911	.886	1	
	Sig.	.000	.000	.000	.000	.000	.000		
	N	117	117	117	113	117	117	117	

NONPAR CORR
  /VARIABLES=MAX AVG STD Ki67# CVprediction Diameter TV
  /PRINT=SPEARMAN ONETAIL NOSIG
  /MISSING=PAIRWISE.


Chart


 [DATA4] /Users/pengmingzheng/Documents/ggo ct/materials and data.sav
